# Enlarged Interior Built Environment Scale Modulates High-Frequency EEG Oscillations

**DOI:** 10.1523/ENEURO.0104-22.2022

**Published:** 2022-09-21

**Authors:** Isabella S. Bower, Gillian M. Clark, Richard Tucker, Aron T. Hill, Jarrad A. G. Lum, Michael A. Mortimer, Peter G. Enticott

**Affiliations:** 1 Cognitive Neuroscience Unit, School of Psychology, Deakin University, Burwood, Victoria 3125, Australia; 2 School of Architecture and Built Environment, Faculty of Science, Engineering and Built Environment, Deakin University, Geelong, Victoria 3220, Australia; 3 School of Engineering, Faculty of Science, Engineering and Built Environment, Deakin University, Geelong, Victoria 3216, Australia; 4 Department of Psychiatry, Central Clinical School, Faculty of Medicine, Nursing & Health Sciences, Monash University, Melbourne, Victoria 3004, Australia

**Keywords:** cave automatic virtual environment, electroencephalography, heart rate variability, respiration, scale, skin conductance

## Abstract

There is currently no robust method to evaluate how built environment design affects our emotion. Understanding emotion is significant, as it influences cognitive processes, behavior, and wellbeing, and is linked to the functioning of physiological systems. As mental health problems are becoming more prevalent, and exposure to indoor environments is increasing, it is important we develop rigorous methods to understand whether design elements in our environment affect emotion. This study examines whether the scale of interior built environments modulate neural networks involved in emotion regulation. Using a Cave Automatic Virtual Environment (CAVE) and controlling for indoor environmental quality (IEQ), 66 adults (31 female, aged 18–55) were exposed to context-neutral enclosed indoor room scenes to understand whether built environment scale affected self-report, autonomic nervous system, and central nervous system correlates of emotion. Our results revealed enlarged scale increased electroencephalography (EEG) power in the β bandwidth. Frontal midline low-γ and high-γ power were also found to increase with enlarged scale, but contrary to our hypothesis, scale did not modulate frontal midline power or lateralization in the θ or α bandwidths. We did not detect an effect of scale on autonomic indicators or self-reported emotion. However, we did find increased range in skin conductance response (SCR) and heart rate variability (HRV) to the built environment conditions. This study provides a rigorous empirical framework for assessing the environmental impact of a design characteristic on human emotion and suggests that measures of high-frequency oscillations may provide a useful marker of the response to built environment.

## Visual Abstract

**Figure:**
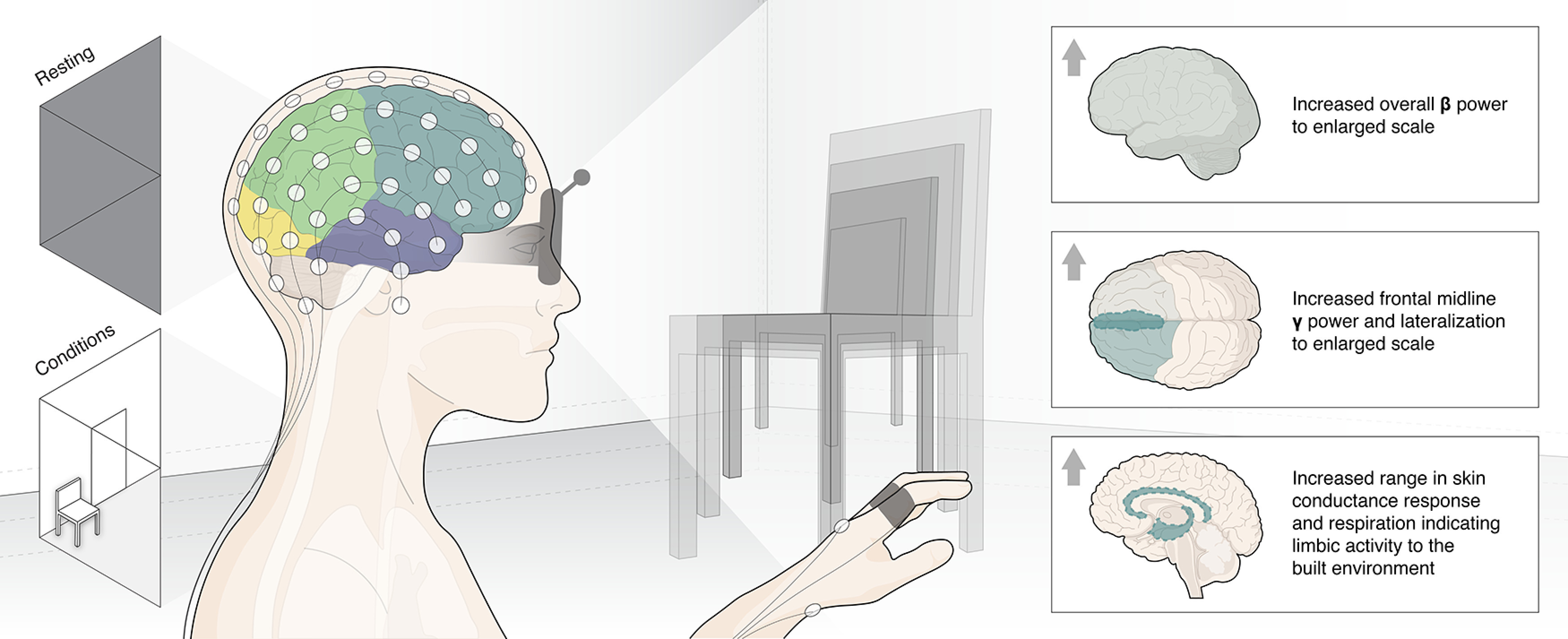

## Significance Statement

Our empirical study provides a technique and approach for assessing the impact of built environment design on emotion. Using virtual reality (VR), we assessed autonomic nervous system, electroencephalography (EEG) correlates and self-report of emotion to built environments that vary in scale. Although we did not detect autonomic and EEG markers linked to emotional processing, we found evidence that enlarged scale of the built environment modulates high-frequency oscillatory activity, which may have further implications for attention and cognitive performance. This novel approach for measuring neural correlates and physiological indicators controlled the exposure through a Cave Automatic Virtual Environment (CAVE), while monitoring indoor environmental quality (IEQ). This research and technique enhance our understanding of how to predict, design, and optimize interior spaces for optimal mental health.

## Introduction

There is currently no robust method to evaluate how building design affects our emotion. Emotion is recognized to play an important role in our mental and physical health (Damasio, 1998; Lopez et al., 2018). Accordingly, understanding whether the buildings we inhabit affect our emotions is critical. Through building design, we may be able to mediate health outcomes, leading to major health and economic benefits for society (Hoisington et al., 2019).

Environmental enrichment studies in animal models have suggested that features of the physical enclosure, including size of the environment (Barker et al., 2017), impact cellular, molecular and behavioral outcomes (Nithianantharajah and Hannan, 2006; Janssen et al., 2018). Despite this, there have been few human studies investigating interior environments as a component of environmental enrichment (McDonald et al., 2018). Following work indicating the role of environmental enrichment on brain structure, function and behavior, we investigated the built environment as one of the enrichment modulating factors; specifically, the scale of enclosure.

Scale has strong theoretical underpinnings in social and architectural history (Raskin, 1954; Alexander et al., 1977). The concept of understanding whether room or enclosure scale affects behavior patterns is not new, with work undertaken in both animal and human studies (Wolfe, 1975). However, commonly research does not distinguish between the concept of physical and social environment. In human studies, “proxemics”, or the behavior and interaction of space and people, is often studied (Evans et al., 1996). Similarly, in animal models the concept of “housing density” is explored (Whittaker et al., 2012). This makes it difficult to determine whether the scale of the physical environment makes a difference, or whether differences result from affordances the scale produces for social interactions.

Emerging empirical studies exploring design characteristics of interior built environments have approached the question using experimental designs where design aesthetics comprise a complex array of features and characteristics (Vartanian et al., 2015; Coburn et al., 2020). However, across this emerging research field, questions exist as to the validity of the experimental design approach and reporting parameters to ensure reproducibility (Bower, 2019).

In this study, we investigated whether the scale of an interior room would result in modulation of autonomic, EEG, and self-report indicators of emotion. We defined emotion as a response to an environmental event involving multiple systems of cognitive, autonomic, and behavioral response (Levenson, 1988; Thayer and Lane, 2000; Hagemann et al., 2003). Here, we tested whether there was a change in participants’ autonomic nervous system response through electrocardiography (ECG), skin conductance response (SCR) and respiration measures; alongside recording central nervous system response with electroencephalography (EEG). Self-reported emotion was assessed using the self-assessment manikin, based on the affective dimensional model classification of emotion. Demographic and personality data were also collected to investigate whether individual factors influenced responses to built environment scale, as existing studies show personality dimensions, such as neuroticism, can affect how individuals interpret and respond to the environment (LeBlanc et al., 2003).

To reduce the complexity of building design, the study used a Cave Automatic Virtual Environment (CAVE), to create an environmentally controlled, cost-effective simulation, and providing greater sensorimotor integration than virtual reality (VR) headsets (Sanchez-Vives and Slater, 2005; Bohil et al., 2011; Kalantari et al., 2021). Scene neutrality was carefully considered through non-context-specific visual cues in the form of a closed door and a chair to help participants determine height, width, and surface depth (Brouwer et al., 2005).

As this field of research is early in development, in conjunction with our a priori hypotheses, we opted to perform exploratory analyses across the remaining EEG power spectra regions of interest (ROIs) and of the overall power spectral density. This approach was selected as we were interested in understanding whether the built environment may affect other cognitive functions such as perception, attention, and memory which have been associated with higher frequency oscillatory activity. Neural oscillations in the γ frequency range have been associated with visual tasks such as perception (Keil et al., 1999), attention (Müller et al., 2000), and memory (Tallon-Baudry et al., 1998). We expected changes to scale would result in increased frontal midline power and frontal hemispheric lateralization in the θ and α bandwidths, because of their association with emotion (Aftanas and Golocheikine, 2001; Coan and Allen, 2004; Davidson, 2004). We also hypothesized scale conditions would increase baseline autonomic measures, and self-report may not reflect underlying autonomic or EEG modulations.

## Materials and Methods

**Figure 1.**
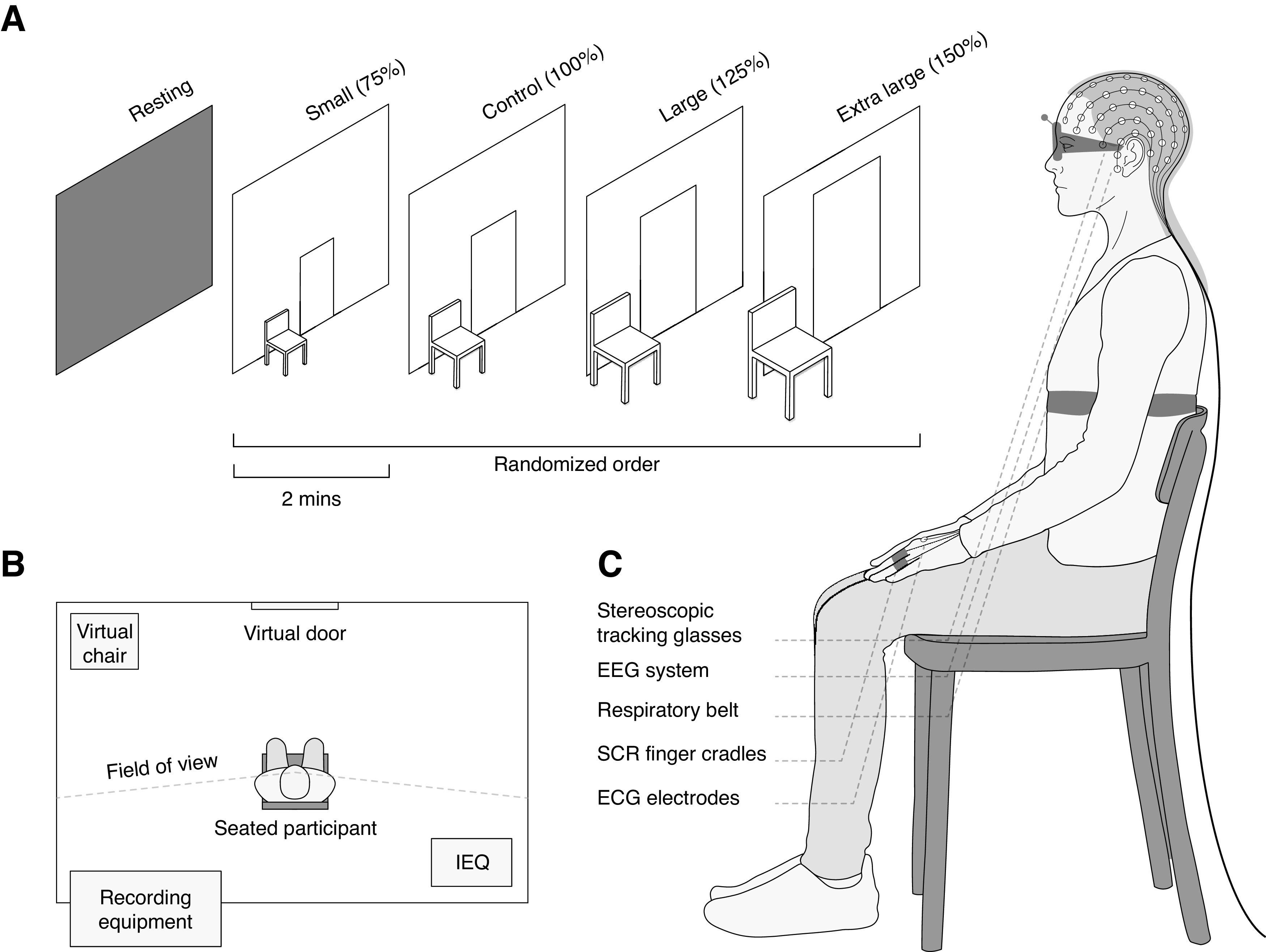
Experimental design and setup. ***A***, Isometric view of conditions. Participants were presented with eyes open resting state, followed by four randomized scale conditions. Each scene lasted 2 min, between which the resting state was displayed while the participant completed a self-report assessment of emotion. ***B***, Floor plan indicating the position of items in the experiment. IEQ variables were measured continuously, and all recording equipment was positioned outside of the participants field of view. ***C***, Diagram of equipment fitted to participant including stereoscopic tracking glasses, EEG system, respiratory belt, SCR finger cradles, and ECG electrodes. Diagrams are representative, not drawn to exact scale.

To investigate our research questions, we examined whether there are detectable differences in autonomic, EEG, and self-report indices of emotion when changing the design characteristic of scale within a virtual built environment. Using EEG, we investigated frontal midline power and lateralization in the α and θ bandwidths. In addition to our primary hypothesis-driven analyses, we conducted exploratory data driven analysis of low-γ and high-γ, and overall power spectral density across electrodes. The study was approved by the Deakin University Human Research Ethics Committee and carried in accordance with relevant guidelines and regulations. On completion of the study participants were offered a $20.00 AUD gift voucher as reimbursement for their time. An overview of the experimental design and setup is illustrated in Figure 1.

### Participants

The sample size for this study was determined by an a priori power analysis using G*Power 3.1.9.3 (Faul et al., 2007). Because of the limited robust studies conducted, a small to moderate effect size was selected (f = 0.15) with one group and five measurements, a power of 0.95, a correlation among repeated measures of 0.6, and nonsphericity correction of 1. This indicated a total sample size of 68 participants would be required.

The study took place over a consecutive five-week period at Deakin University Waurn Ponds Campus, Geelong, Victoria, Australia. We recruited 66 adults (31 women, mean age = 34.9 ± 11.3 years, four participants left-handed) aged between 18 and 55 years old; with no prior training or work experience in built environment design; and with no prior diagnosed psychiatric, neurologic, or neurodevelopmental conditions. A healthy adult sample was selected because of the experimental nature of the study and to reduce confounding variables. All participants were able to speak and read English and had normal or corrected-to-normal vision. 12 different languages were spoken at home by participants and 14 countries were identified as the location participants spent the most time growing up in.

### Procedure

Participants were individually tested, and each session ran for ∼90 min. On arrival, participants completed a secure web-based survey (Qualtrics), which took between 15 and 30 min. The survey included 13 questions regarding socio-demographic background, experience, and expertise for both VR and computer gaming. This was followed by a personality test using the abbreviated International Personality Inventory Pool (IPIP-NEO-120; Johnson, 2014). The open-source test included 24 questions across the five-factor-model domains of openness, conscientiousness, extroversion, agreeableness and neuroticism (OCEAN) (McCrae and John, 1992). Once the participant completed the self-report survey, the researcher explained the equipment to be used and demonstrated how this would be fitted. To reduce external factors that could influence physiological measures, we asked whether participants had eaten before the experiment and prompted the opportunity to use the bathroom before the experiment to avoid bladder discomfort (Quintana and Heathers, 2014).

Before fitting electrophysiological equipment, skin surfaces on hand and wrist sites which would be in contact with ECG electrodes and the SCR cradles were cleaned to remove any residues. This was done by using a cotton tip to rub the skin surface with an abrasive gel (Weaver and Company NuPrep) and cleaning the surface of any residue with an alcohol wipe. Three ECG electrodes were placed on the hand and wrists. The positive electrode was located on the left wrist and the negative electrode was placed on the right wrist. The reference was placed on the knuckle of the middle finger on the left-hand side. For SCR, finger electrodes were placed underneath the middle and index finger on the left hand and secured with a Velcro strap. A respiratory belt transducer was positioned on the sternum and secured firmly around the chest. A 10 min, three-lead ECG recording (PowerLab, LabChart Pro 8.1.16) was performed. Circuit zero was applied before the first recording and a subject zero was undertaken between each condition recording. A sampling rate of 1000 Hz and notch filter of 50 Hz was used.

We used a 64-channel cap (Philips Hydrocel Geodesic Sensor Net 64-channel HCGSN) for acquiring EEG data. Net Station 5 Geodesic EEG software, version 5.4.2 (Electrical Geodesics Inc) was used to record EEG data. The cap was positioned on the head after being soaked in an electrolyte solution. Data were acquired at a sampling rate of 1000 Hz, with Cz as the online reference. The Cz electrode was not included in our analysis, however, for the purposes of visualization, we have interpolated the Cz site for figures. A continuous recording was created for each participant and the EEG trace was manually time stamped by the researcher at the start and end of each 2-min scene exposure. The majority of impedances were kept under 50 kΩ with an average value of 25.2 kΩ (SD = 7.75).

Participants were then led into the VR lab containing the CAVE. Participants were assisted to step into the CAVE and take a seat while the researcher carefully took the cords leading from the attached EEG, ECG, and SCR sensors to connect to the monitoring equipment behind the participant. A pair of stereoscopic glasses to view the CAVE projection were then carefully fitted on top of the EEG cap. These remained on throughout the experiment. Impedances were checked and adjusted when necessary to ensure the quality of electrode-to-scalp contact.

Participants were seated for the duration of the experiment and instructed to pay attention to the scene they were presented. We elected to run a static resting state study where participants sat immersed in the space, rather than setting a task involving movement through thresholds, as this has been thought to affect cognition and memory (Pettijohn and Radvansky, 2016). By sitting, any height differences were also minimized between participants, and this also helped to minimize any movement-related artefacts in the electrophysiological measures. Each participant was exposed to an eyes-open resting state, followed by four built environment scenes in randomized order, displayed for 2 min each. At the end of each scene the virtual environment was returned to the resting state scene and the participant was asked to complete a short self-report survey using a five-point visual self-assessment manikin. This process was repeated five times, with a total duration of ∼15–20 min.

We measured indoor environmental quality (IEQ) variables within the CAVE throughout the study. These measures were analyzed to ensure any fluctuations to these properties linked to data which may influence emotion and neurophysiological response were minimized. To reduce the chance of negative influence all data were collected over a consecutive period in spring to reduce heat/cool load on the building triggering changes in the heating, ventilation, and air conditioning system.

### Equipment and stimuli

#### CAVE

The CAVE consisted of three walls (3 m wide × 2.4 m high) and a floor (2.4 m wide × 3 m long), each with Barco Galaxy NW-12 stereoscopic projectors. The projectors connect to a series of image generators (computers) each consisting of Nvidia Quadro P6000 graphic cards. The graphic cards are synced using Quadro Sync II cards at 120 Hz (60 Hz per eye) to frame lock the projectors to ensure rendered images are displayed at the same time. The CAVE uses an optical-based tracking system consisting of eight cameras that tracks active LED markers located on the stereoscopic glasses to track user movements. The tracking system operates at 240 Hz with sub millimeter accuracy and connects back to a Virtual Reality Peripheral Network (VRPN) server. The CAVE uses a custom-built Unity environment to run VR experiences with Vertical Sync (VSync) set to 60 frames per second. The Unity environment connects to the tracking systems via VRPN server using an ethernet connect and updates the tracked position on each rendered frame.

#### Virtual environment development and CAVE integration

Autodesk Revit was used to create a 3D model that represented a conventional cubic room that was then exported into the Unity game engine (2019.2.15) for CAVE integration. A matte plaster texture was applied to the three wall surfaces with a slight gloss texture of bumpy concrete applied to the floor. A matte wood texture was applied to the door, doorway and chair with a low gloss metal surface applied to the door handle. Once material color, texture settings and lighting had been applied to the model, the room was duplicated (Unity Prefabs) into three separately scaled rooms. Prebaked lightmaps were applied for each scaled room to ensure consistent lighting and texture relative to the scale and “realistic” as possible to view.

The control condition was designed using Standards Australia measurements for a residential internal door (820 × 35 × 2040 mm) (Standards Australia 2017), and room dimensions were modelled of the physical CAVE walls (3200 × 3200 × 2400 mm). For neutrality, the resting state scene (no built environment) was rendered in black [R0, G0, B0, hue (degrees) = 0, saturation (%) = 0, brightness (%) = 0]. As a result of the white finish of the projector screens, this black virtual background appears as a dark gray when displayed on the screens. All scale conditions were rendered with a white finish [R255, G255, B255, hue (degrees) = 0, saturation (%) = 0, and brightness (%) = 100, and smoothness = 50%]. The scale variables included a “small” condition where the room size was reduced to 75% and two conditions where the room was enlarged by 125% “large” and 150% “extra-large” compared with the 100% control.

#### Room configuration and setup

A wooden fixed chair with a seat pad for comfort and back support for posture consistency was positioned in the center of the CAVE, effectively within the center of each virtual room regardless of scale. The chair remained in the central position to ensure all participants were situated in the same location. Room lights were switched on for safety when a participant entered the CAVE, that displayed the resting state scene. After the participant was set up and briefed on the experiment procedure, the researcher turned off the room lights.

#### IEQ

CR100 Measurement and Control System with LoggerNet 4.6.2 software (Campbell Scientific, Inc) was used to acquire and record data. Before the experiment, we completed a test recording and calibrated the recording equipment to ensure the readings were accurate in accordance with EN ISO 7730 Fanger Comfort Model (Fanger, 1970).

IEQ data were recorded at 1 min intervals which were date and time stamped. We averaged the 1 min readings from the corresponding time stamped data within each participants session to create an overall average per person and then determined the average across all participants. Although the VR lab was acoustically soundproof and no talking occurred during the scene recordings, a handheld sound level meter was used to capture fluctuating mechanical equipment noises from the CAVE projector lamp ventilation and cooling system which could not be controlled. Sound level recordings were conducted at different intervals during experiments to establish an overall range across the five-week period. Overall mean air and wet-bulb globe temperature was within the 21–25°C range for optimal performance (Seppänen and Fisk, 2006), the carbon dioxide concentration throughout the testing period was within the indoor air concentration range of 500–1500 ppm, and the mean relative humidity was under 50% (Seppänen and Fisk, 2004). Sound pressure levels were also within an accepted range for the experiment (Basner et al., 2014).

#### Self-report data

Self-report of emotion was collected using the self-assessment manikin where three dimensions, pleasure, arousal, and dominance, are recorded by the participant using a visual 5-point scale (Bradley and Lang, 1994; Mehrabian, 1996). The participant used an iPad to complete the self-report using a Qualtrics survey at the end of each stimulus. No time limit was given for the self-evaluation, and the researcher remained outside of the CAVE until the participant verbally signaled they had completed the evaluation.

### Data analysis

#### Physiological data

Physiological data were acquired using PowerLab 4/35 (ADI Instruments PL3504) with a respiratory belt transducer (ADI Instruments TN1132/ST), Ag/AgCl ECG electrodes (Ambu Bluesensor N) and SCR finger plate electrodes (ADI Instruments MLT118F). Data for all physiological measures were acquired at 1000 Hz, and for SCR circuit zero was applied before the first recording and a subject zero was undertaken between each condition recording. Online filtering parameters differed between measures: ECG −100 to 100 mV; SCR −40 to 40 μS; and respiration −10 to 10 V. Five channels were set to record and calculate ECG, SCR and respiration. Results were divided into time segments (10–60, 60–110 s) and one overall time block (10–110 s) to capture whether an effect occurred at onset but diminished because of habituation over the recording. Three datasets from participants were excluded in the SCR and respiration analysis because of equipment fault. In respiratory data, 10 s from the onset of recording was removed for the measure to be accurately detected. For consistency the last 10 s was also removed.

Heart rate variability (HRV) settings used a beat classification for RR intervals between 600 to 1400 ms and complexity of 1 to 1.5. Ectopic heartbeats were excluded from analysis. Detection was adjusted to a minimum peak height of 1.2 SD and typical QRS width between 80 ms over a 350 ms minimum period. A low-pass filter of 30 Hz was used. We analyzed the root mean square successive difference (RMSSD) and the SD of the R-R interval (SDRR) time domain components of the QRS complex within the ECG recording in accordance with the Task Force of the European Society of Cardiology and the North American Society of Pacing and Electrophysiology (Camm et al., 1996). Respiration frequency was measured using the cyclic measurements function with scoring parameters of 1.3 SD threshold for detecting minimum peak height. To accommodate the time lag in the equipment detecting the first breath after recording, the first 7 s and last final 7 s of the continuous file for each participant across conditions was removed. Because of technical issues in the recording, two files did not record correctly and were excluded from analysis.

We collected HRV through time-domain, frequency-domain, and nonlinear measurements. Data were analyzed using RStudio (version 1.3.959). *N* = 2 HRV and breathing datasets were excluded from the analysis because of HRV arrythmia; however, data for SCR were still incorporated. To correct for distribution, a log transformation (log10) was applied to both HRV and SCR data. To correct for normality, we removed outliers which fell below [Q1 – (1.5 × IQR)] and were above [Q3 + (1.5 × IQR)]. A within subjects repeated measures ANOVA with the Greenhouse–Geisser correction for sphericity was used across the six physiological measures we analyzed. To control for multiple comparisons, a false discovery rate (FDR) correction was applied to the results (Benjamini and Hochberg, 1995).

#### EEG

The EEG data were preprocessed using EEGLab (v2019.1) (Delorme and Makeig, 2004), an open source graphic user interface and toolbox plugin for MATLAB R2019b (v9.7.0.1471314, MathWorks, Inc). We applied a bandpass filter from 1 to 70 Hz (zero-phase Butterworth filter) on continuous EEG data. A 47- to 53-Hz notch-filter was applied to exclude electrical interference from the CAVE environment. We then removed eye channels and the Cz reference channel. Next, we rejected channels if the kurtosis value was >5 SDs outside the average and replaced information in those channels using a spherical spline interpolation. Data were subsequently re-referenced to the average of all electrodes. To aid the removal of recording noise we applied the SOUND algorithm using input parameters of five iterations to evaluate noise in each channel and 0.2 regularization level (λ value) to control the amount of cleaning (Mutanen et al., 2018). Each participant’s continuous EEG data were decomposed using independent component analysis (FastICA algorithm; Hyvärinen and Oja, 2000), with artifactual components identified with assistance from the ICLabel plugin (Pion-Tonachini et al., 2019). A component was removed if ICLabel classified the probability of that component containing brain data were <30% and the component was not in the “other” category. The mean of the components removed for each subject was 6.16 ± 3.92.

Using the time-stamped event markers in the continuous recording, each file was then split into 120 s block files using the start marker for each condition. Data were segmented into 3-s epochs for subsequent analyses. Finally, additional artifact rejection was performed to remove any remaining noisy epochs with data exceeding ± 150 μV using the EEGLab ‘pop_eegthresh’ function. After cleaning we calculated the average epochs remaining for each condition and participant (mean number of epochs = 39.5 ± 1.46). Lastly, we converted data from each participant/electrode to the frequency domain using the fast Fourier transform (FFT) with Hanning taper in the FieldTrip toolbox for EEG/MEG-analysis (1-Hz frequency steps between 1 and 70 Hz; Oostenveld et al., 2011).

To calculate power in the different frequency bands, we created averages across each separate frequency band for each electrode: δ (1–3 Hz), θ (4–7 Hz) α (8–12 Hz), β (13–29 Hz), low-γ (30–45 Hz), and high-γ (55–70 Hz). Power was then averaged over electrodes within three hypothesis-driven a priori ROIs: frontal midline (AFz, Fz, FCz), frontal right-hemispheric (F10, F8, AF4, F6, FT8, F2, F4, FC6, FC4, and FC2), and frontal left-hemispheric sites (F9, F7, AF3, F5, FT7, F1, F3, FC5, FC3, and FC1). During a posteriori analysis of γ lateralization, we selected sites from across the whole scalp to run an exploratory analysis (F3-F4, FT7-FT8, FC5-FC6, FC3-FC4, C3-C4, C5-C6, TP7-TP8, CP5-CP6, P7-P8, P9-P10). A lateralization index was generated to understand the power difference between the average over the frontal left and right ROIs, where higher values correspond to stronger power in the right compared with the left ROIs (Demaree et al., 2005):

(α)=(α′′(right)′′−α′′(left)′′)/(α′′(left)′′+α′′(right)′′).

For statistical tests, we removed values that caused the violation of normality assumptions (according to the Shapiro–Wilk test). We removed extreme values which fell below [Q1 – (1.5 × IQR)] and were above [Q3 + (1.5 × IQR)]. Overall statistical analysis was conducted in RStudio using a repeated measures ANOVA with G-G correction. To correct for multiple comparisons where significance was detected within-subjects, the FDR method was used (Benjamini and Hochberg, 1995). The FDR is an alternative approach to multiple testing which increases detection power over traditional methods for multiple testing (Genovese, 2015).

### Code accessibility

Source data and analysis code to accompany this manuscript submission are all available to be viewed on Open Science Framework: https://doi.org/10.17605/OSF.IO/5MVN3.

## Results

### Overview

Six measures were preselected to analyze physiological response to robustly compare group differences in distribution, variability and skew (Rousselet et al., 2017). We calculated the power spectra of the five EEG frequency bands averaged across participants for each condition. Hypothesis driven a priori analyses for EEG data included increased right frontal α and θ band lateralization (Coan and Allen, 2004; Davidson, 2004) and increased frontal α and θ midline power (Aftanas and Golocheikine, 2001). Studies have indicated that lower α and θ power in the left than right hemisphere is associated with positive emotion, while lower power in the right than left hemisphere can be seen for negative emotion (Ahern and Schwartz, 1985; Demaree et al., 2005). Self-report rating changes were compared with ± direction of the physiological and EEG responses, to determine whether the pattern of the two measurement types aligned. On inspection of the extracted raw data, an exploratory test was run a posteriori to analyze γ frontal midline power and lateralization, alongside overall power spectral density across bandwidths for completeness. Participant socio-demographic and personality data were also reviewed a posteriori to understand whether underlying characteristics in the study sample interacted with the themes emergent in the results. No significant effect was found, see Extended Data Figure 2-2.

### Increased power spectral density was found in the β bandwidth to enlarged scale

We found significant differences between the scale conditions for β power across the average of all channels (*F*_(4,201)_ = 7.04, *p* ≤ 0.001, *η*^2^_p_ = 0.110). Power was significantly lower in resting state than: small [M_diff_ = –0.041, SE_diff_ = 0.015, *t*_(57.0)_ = −2.808, *p*_corrected_ = 0.016, 95% CI (–0.068, –0.010)], control [M_diff_ = –0.042, SE_diff_ = 0.013, *t*_(57.0)_ = −3.101, *p*_corrected_ = 0.015, 95% CI (–0.069, –0.015)], large [M_diff_ = –0.036, SE_diff_ = 0.036, *t*_(57.0)_ = −2.729, *p*_corrected_ = 0.016, 95% CI (–0.066, –0.013)], and extra-large [M_diff_ = −067, SE_diff_ = 0.015, *t*_(57.0)_ = −3.820, *p*_corrected_ ≤ 0.001, 95% CI (–0.095, –0.040)]. There was also significant increase in power when comparing the small to the extra-large condition [M_diff_ = −027, SE_diff_ = 0.012, *t*_(57.0)_ = −2.217, *p*_corrected_ = 0.044, 95% CI (–0.048, –0.002)], the control to the extra-large [M_diff_ = 0.006, SE_diff_ = 0.013, *t*_(57.0)_ = 0.445, *p*_corrected_ = 0.018, 95% CI (–0.046, –0.008)], and the large to the extra-large [M_diff_ = –0.032, SE_diff_ = 0.011, *t*_(57.0)_ = −2.788, *p*_corrected_ = 0.016, 95% CI (–0.046, –0.003)].

In the low-γ bandwidth we found significant differences (*F*_(4,161)_ = 13.6, *p* ≤ 0.001, *η*^2^_p_ = 0.229). During *post hoc* analysis we detected significantly lower power for resting state to: small [M_diff_ = –0.184, SE_diff_ = 0.027, *t*_(46.0)_ = −5.409, *p*_corrected_ ≤ 0.001, 95% CI (–0.200, –0.100)], control [M_diff_ = –0.128, SE_diff_ = 0.026, *t*_(46.0)_ = −4.918, *p*_corrected_ ≤ 0.001, 95% CI (–0.177, –0.076)], large [M_diff_ = –0.129, SE_diff_ = 0.030, *t*_(46.0)_ = −4.378, *p*_corrected_ ≤ 0.001, 95% CI (–0.188, –0.076)], and extra-large [M_diff_ = –0.173, SE_diff_ = 0.025, *t*_(46.0)_ = −6.934, *p*_corrected_ ≤ 0.001, 95% CI (–0.222, –0.131)]. We also detected a significant increase from the control to the extra-large in the low-γ bandwidth, but this was lost after applying FDR correction for multiple comparisons. Lastly, an increase in high-γ power was detected in scale conditions when compared with the resting state (*F*_(4,160)_ = 12.8, *p* ≤ 0.001, *η*^2^_p_ = 0.217). These effects were only seen between resting and the scale conditions: small ([M_diff_ = –0.198, SE_diff_ = 0.040, *t*_(46.0)_ = −4.949, *p*_corrected_ ≤ 0.001, 95% CI (–0.291, –0.145)], control [M_diff_ = –0.144, SE_diff_ = 0.037, *t*_(46.0)_ = −3.905, *p*_corrected_ ≤ 0.001, 95% CI (–0.222, –0.078)], large [M_diff_ = –0.189, SE_diff_ = 0.040, *t*_(46.0)_ = −4.708, *p*_corrected_ ≤ 0.001, 95% CI (–0.243, –0.084)], and extra-large [M_diff_ = –0.223, SE_diff_ = 0.035, *t*_(46.0)_ = −6.382, *p*_corrected_ ≤ 0.001, 95% CI (–0.276, –0.139)].

**Figure 2.**
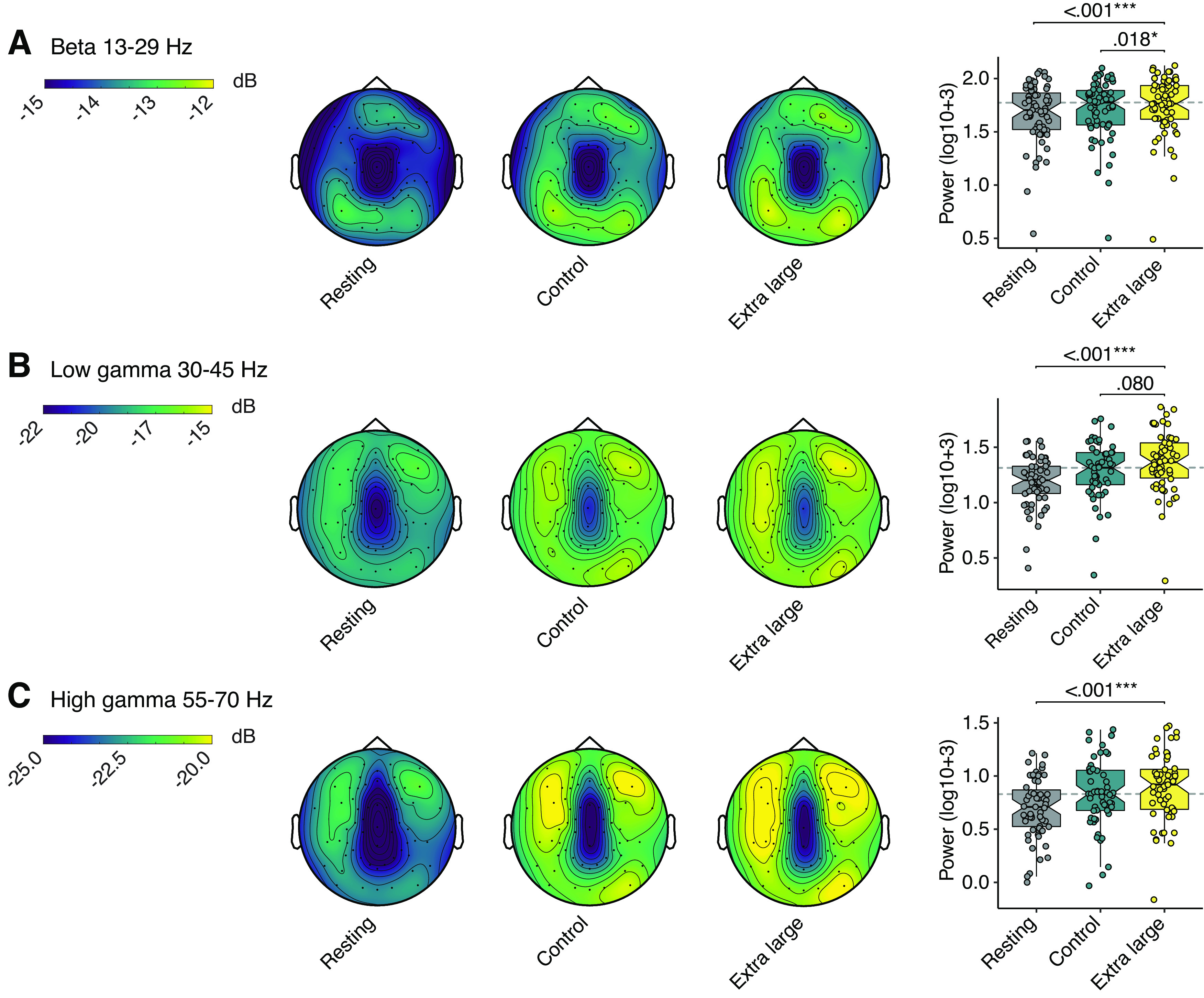
Significant differences between the control and extra-large condition for EEG power spectral density was found in the β bandwidth. To illustrate the differences, we have plotted EEG topographies and boxplots with quartile ranges and medians for the overall power spectra in the β, low-γ, and high-γ bandwidths. Note the Cz site has been interpolated for this figure. β 13–29 Hz (***A***), low-γ 30–45 Hz with amplitude range (***B***), and high-γ 55–70 Hz (***C***).

We also detected significant differences in the remaining bandwidths; however, *post hoc* analysis revealed these differences were contained between the resting state and built environment scale conditions. This included the δ bandwidth (*F*_(3,158)_ = 15.1, *p* ≤ 0.001, *η*^2^_p_ = 0.229). With differences between resting and the scale conditions: small ([M_diff_ = –0.153, SE_diff_ = 0.031, *t*_(51.0)_ = −5.001, *p*_corrected_ ≤ 0.001, 95% CI (–0.206, –0.086)], control [M_diff_ = –0.134, SE_diff_ = 0.029, *t*_(51.0)_ = −4.598, *p*_corrected_ ≤0.001, 95% CI (–0.189, –0.072)], large [M_diff_ = –0.132, SE_diff_ = 0.029, *t*_(51.0)_ = −4.578, *p*_corrected_ ≤ 0.001, 95% CI (–0.185, –0.076)], and extra-large [M_diff_ = –0.173, SE_diff_ = 0.031, *t*_(51.0)_ = −5.628, *p*_corrected_ ≤ 0.001, 95% CI (–0.226, –0.106)]. Similar effects were seen in the θ bandwidth (*F*_(3,164)_ = 13.0, *p* ≤ 0.001, *η*^2^_p_ = 0.203). Follow-up analysis indicated lower power was detected for resting state than small [M_diff_ = –0.093, SE_diff_ = 0.021, *t*_(51.0)_ = −4449, *p*_corrected_ ≤ 0.001, 95% CI (–0.131, –0.049)], control [M_diff_ = –0.083, SE_diff_ = 0.020, *t*_(51.0)_ = −4.161, *p*_corrected_ ≤ 0.001, 95% CI (–0.125, –0.047)], large [M_diff_ = –0.095, SE_diff_ = 0.018, *t*_(51.0)_ = −5.269, *p*_corrected_ ≤ 0.001, 95% CI (–0.137, –0.063)], and extra-large [M_diff_ = –0.110, SE_diff_ = 0.021, *t*_(51.0)_ = −5.219, *p*_corrected_ ≤ 0.001, 95% CI (–0.155, –0.074)]. Lastly, α waves, which are commonly found during awake rest, showed within-subject effects (*F*_(2,111)_ = 5.00, *p *=* *0.007, *η*^2^_p_ = 0.089); however, follow-up analysis indicated that resting state α power was only significantly lower to the control condition [M_diff_ = 0.083, SE_diff_ = 0.026, *t*_(51.0)_ = 3.178, *p*_corrected_ ≤ 0.001, 95% CI (0.034, 0.136)]. Results are shown in Figure 2. Additional bandwidths are also presented in Extended Data Figure 2-3. Descriptives and significance values for all EEG power spectra are presented in Extended Data Figures 2-1 and 2-2.

10.1523/ENEURO.0104-22.2022.f2-1 Extended Data Figure 2-1 Descriptives (mean/SD) for EEG analysis. Download Figure 2-1, DOCX file.

10.1523/ENEURO.0104-22.2022.f2-2 Extended Data Figure 2-2 Statistical significance values for EEG analysis. Note: power spectral density, frontal midline, and θ and α lateralization statistics are derived from parametric one-way repeated measures ANOVAs. *p*,* p* value; FDR, FDR correction. Download Figure 2-2, DOCX file.

**Figure 3.**
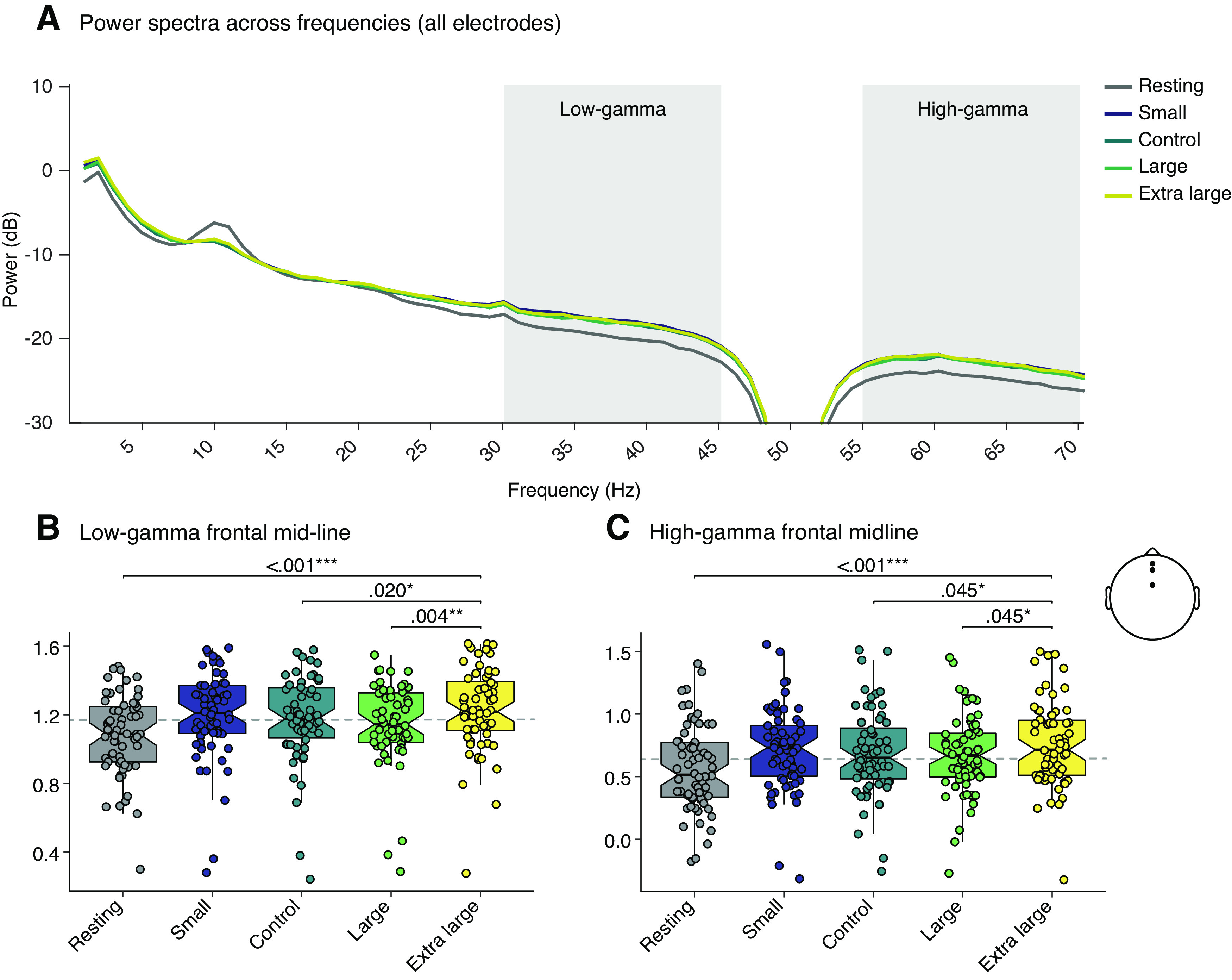
Significant differences in EEG low-γ frontal midline power were found. ***A***, Power spectra plot showing power (dB) across frequencies. The low-γ bandwidth (30–45 Hz) and high-γ bandwidth (55–70 Hz) are highlighted with the gray shading box. The dip represents the 47- to 53-Hz notch filter applied to remove electrical interference from the CAVE environment. ***B***, ***C***, Boxplots with quartile ranges and medians to show increased γ midline power spectra. Each data point overlaid represents a participant’s averaged response from the 2-min exposure.

10.1523/ENEURO.0104-22.2022.f2-3 Extended Data Figure 2-3 EEG topographies and boxplots with quartile ranges and medians for the overall power spectra in the δ, θ, and α bandwidths. Note: the Cz site has been interpolated for this figure. δ 1–3 Hz (***A***), θ 4–7 Hz with amplitude range (***B***), and α 8–12 Hz (***C***). Download Figure 2-3, EPS file.

### Enlarged scale increased frontal midline power and lateralization in the γ bandwidth

An exploratory analysis to further investigate frontal midline and lateralization in the low-γ and high-γ bandwidth was undertaken after analyzing the results of the overall power spectral density. Frontal midline power in the low-γ bandwidth increased with the scale of the room (*F*_(4,207)_ =25.7, *p* ≤ 0.001, *η*^2^_p_ = 0.255). *Post hoc* comparisons showed significant differences between resting state and all conditions: small [M_diff_ = –0.107, SE_diff_ = 0.019, *t*_(53.0)_ = −5.737, *p*_corrected_ ≤ 0.001, 95% CI (–0.147, –0.076)], control [M_diff_ = –0.099, SE_diff_ = 0.018, *t*_(53.0)_ = −5.552, *p*_corrected_ ≤ 0.001, 95% CI (–0.135, –0.066)], large [M_diff_ = –0.082, SE_diff_ = 0.018, *t*_(53.0)_ = −4.488, *p*_corrected_ ≤ 0.001, 95% CI (–0.118, –0.046)], and extra-large [M_diff_ = –0.139, SE_diff_ = 0.017, *t*_(53.0)_ = −8.100, *p*_corrected_ ≤ 0.001, 95% CI (–0.173, –0.108)]. We also detected an increase in power from the control to the extra-large [M_diff_ = –0.046, SE_diff_ = 0.016, *t*_(64.0)_ = −2.882, *p*_corrected_ = 0.020, 95% CI (–0.070, –0.012)], and the large to the extra-large [M_diff_ = –0.031, SE_diff_ = 0.014, *t*_(64.0)_ = −2.180, *p*_corrected_ = 0.004, 95% CI (–0.090, –0.023)].

An effect of condition was also seen in the high-γ bandwidth (*F*_(4,232)_ =16.6, *p* ≤ 0.001, *η*^2^_p_ = 0.211). *Post hoc* analysis revealed differences between the resting state and all conditions: small [M_diff_ = –0.164, SE_diff_ = 0.028, *t*_(62.0)_ = −5.861, *p*_corrected_ ≤ 0.001, 95% CI (–0.211, –0.100)], control [M_diff_ = –0.137, SE_diff_ = 0.025, *t*_(62.0)_ = −5.454, *p*_corrected_ ≤ 0.001, 95% CI (–0.188, –0.089)], large [M_diff_ = –0.132, SE_diff_ = 0.029, *t*_(62.0)_ = −4.543, *p*_corrected_ ≤ 0.001, 95% CI (–0.185, –0.069)], and extra-large [M_diff_ = –0.192, SE_diff_ = 0.025, *t*_(62.0)_ = −7.590, *p*_corrected_ ≤ 0.001, 95% CI (–0.239, –0.141)]. We also found a difference between the control to extra-large [M_diff_ = –0.055, SE_diff_ = 0.026, *t*_(62.0)_ = −2.263, *p*_corrected_ = 0.045, 95% CI (–0.103, –0.007)], and large to extra-large [M_diff_ = –0.060, SE_diff_ = 0.026, *t*_(62.0)_ = −2.320, *p*_corrected_ = 0.045, 95% CI (–0.113, –0.010)].

An effect of condition on frontal midline power was found in the θ band (*F*_(4,181)_ = 9.23, *p* ≤ 0.001, *η*^2^_p_ = 0.156). *Post hoc* comparisons showed these differences were constrained to comparisons between the resting state to conditions, with a significant increase between resting state and all conditions: small [M_diff_ = –0.094, SE_diff_ = 0.023, *t*_(50.0)_ = −4.162, *p*_corrected_ ≤ 0.001, 95% CI (–0.138, –0.052)], control [M_diff_ = –0.087, SE_diff_ = 0.021, *t*_(50.0)_ = −4.140, *p*_corrected_ ≤ 0.001, 95% CI (–0.128, –0.045)], large [M_diff_ = –0.093, SE_diff_ = 0.021, *t*_(50.0)_ = −4.470, *p*_corrected_ ≤ 0.001, 95% CI (–0.133, –0.051)], and extra-large [M_diff_ = –0.103, SE_diff_ = 0.022, *t*_(60.0)_ = −4.711, *p*_corrected_ ≤ 0.001, 95% CI (–0.146, –0.067)]. We also detected an effect for α frontal midline power (*F*_(3,169)_ =8.58, *p* ≤ 0.001, *η*^2^_p_ = 0.118). However, these effects were limited to comparisons between resting state to the built environment scale conditions, which were lost during correction for multiple comparisons.

Significant differences were also detected in the frontal hemispheric θ lateralization (*F*_(3,145)_ = 10.2, *p* ≤ 0.001, *η*^2^_p_ = 0.178). *Post hoc* analysis revealed the significant increases in θ lateralization was between resting state and the scale built environment conditions: small [M_diff_ = –0.033, SE_diff_ = 0.008, *t*_(47.0)_ = 4.092, *p*_corrected_ ≤ 0.001, 95% CI (–0.046, –0.016)], control [M_diff_ = –0.032, SE_diff_ = 0.008, *t*_(47.0)_ = 4.014, *p*_corrected_ ≤ 0.001, 95% CI (–0.047, –0.015)], large [M_diff_ = –0.035, SE_diff_ = 0.008, *t*_(47.0)_ = 4.339, *p*_corrected_ ≤ 0.001, 95% CI (–0.047, –0.017)], and extra-large [M_diff_ = –0.035, SE_diff_ = 0.008, *t*_(47.0)_ = 4.327, *p*_corrected_ ≤ 0.001, 95% CI (–0.044, –0.015)]. We also detected difference in frontal α lateralization (*F*_(3,124)_ = 3.71, *p *=* *0.018, *η*^2^_p_ = 0.072). *Post hoc* analysis revealed there were differences between resting state and the conditions, but these did not survive correction. Results are shown in Figure 3. Descriptives and significance values for EEG frontal midline power and frontal hemispheric lateralization are presented in Extended Data Figures 2-1 and 2-2.

### Autonomic response between resting state and the conditions were found, but not to variations in scale

HRV within-subjects effects for time-domain showed an effect of condition in the RMSSD (*F*_(3,198)_ = 3.89, *p *=* *0.007, *η*^2^_p_ = 0.064). RMSSD reflects changes to vagal tone and is less affected by changes in respiration (Shaffer and Ginsberg, 2017). Follow-up analysis indicated that resting state showed some differences with the scale conditions, but there was not a significant difference between the levels of scale. Specifically, RMSSD resting state values were significantly lower than control [M_diff_ = 0.050, SE_diff_ = 0.015, *t*_(57.0)_ = 3.395, *p*_corrected_ = 0.010, 95% CI (0.020, 0.079)], and the extra-large [M_diff_ = 0.040, SE_diff_ = 0.014, *t*_(57.0)_ = 2.819, *p*_corrected_ = 0.035, 95% CI (0.013, 0.069)], but we did not detect significant difference to the small or large conditions. We detected an effect in the SDRR (*F*_(4,205)_ = 2.79, *p *=* *0.032, *η*^2^_p_ = 0.047). However, *post hoc* analysis revealed these values did not survive correction for multiple comparisons.

Respiration measures analyzed were the mean value and maximum minus minimum (Mx-Mn). Within-subjects comparisons for the mean (*F*_(3,135)_ = 2.22, *p *=* *0.096, *η*^2^_p_ = 0.042) and Mx-Mn (*F*_(3,138)_ = 1.07, *p *=* *0.368, *η*^2^_p_ = 0.024) did not reveal significant differences between conditions.

**Figure 4.**
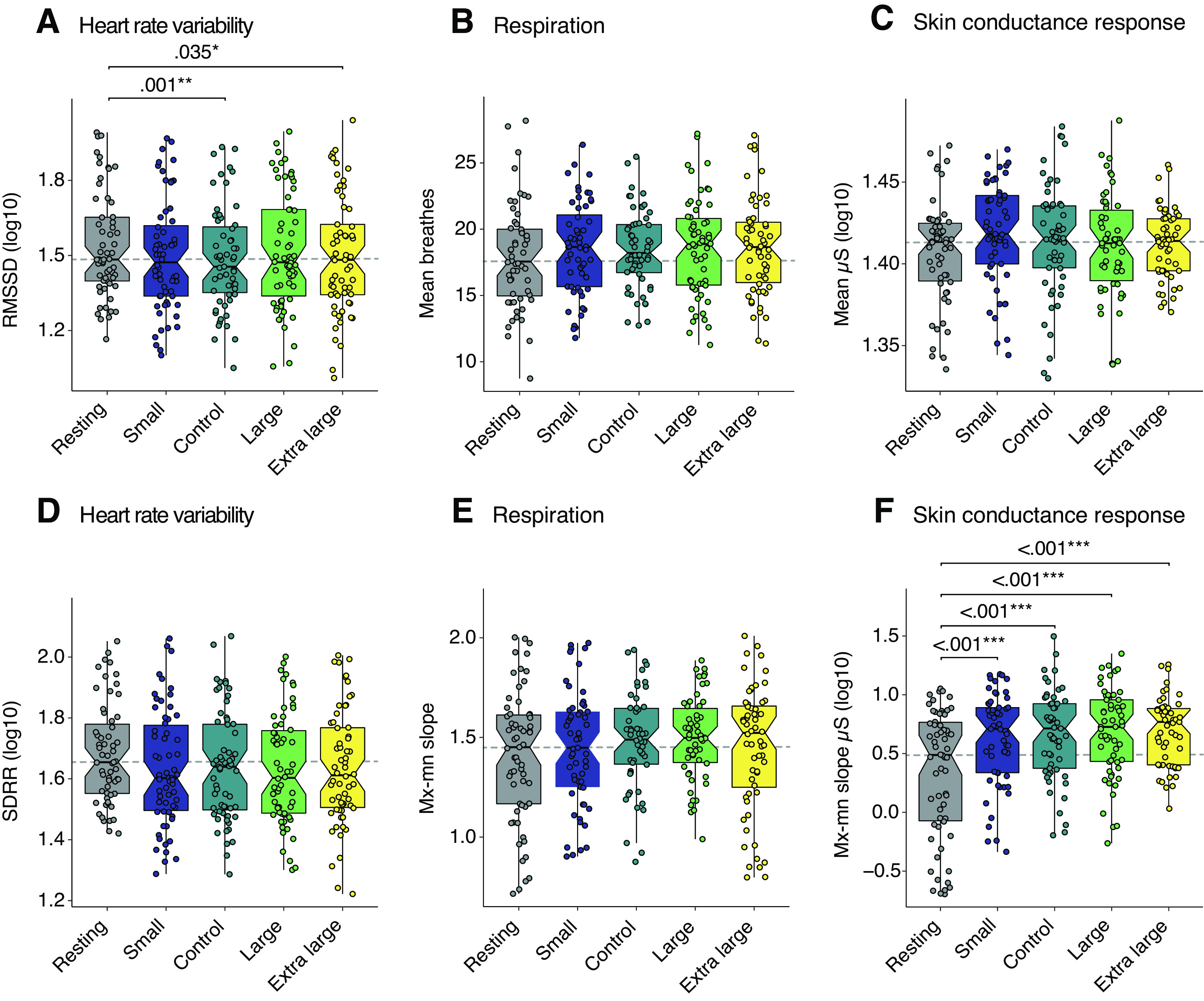
Physiological measures between the resting-state, small, control, large, and extra-large conditions. ***A–F***, Boxplots with quartile ranges and medians for physiological measures analyzed using raw values. Each data point represents a participant’s averaged response from the 2-min exposure. Significance values (FDR-corrected) from the data after transform and removal of outliers have been superimposed to indicate where significant differences were found. All participants were exposed to the resting state first, before the randomized conditions. We did not detect a difference between the control and scale conditions; however, significant differences were detected between the resting-state and built environment scale conditions were found in measures analyzing the change in range, such as maximum–minimum slope for SCR and the RMSSD for HRV.

SCR measures were the mean and the Mx-Mn of the slope. The within-subjects analysis did not show significant differences between conditions in the mean (*F*_(3,115)_ = 1.68, *p *=* *0.170, *η*^2^_p_ = 0.046). There was, however, a significant difference between conditions in Mx-Mn (*F*_(3,150)_ = 10.7, *p* ≤ 0.001, *η*^2^_p_ = 0.171). *Post hoc* comparisons for the Mx-Mn showed a significant increase from resting state to conditions with small [M_diff_ = –0.234, SE_diff_ = 0.059, *t*_(57.0)_ = −3.992, *p*_corrected_ = 0.002, 95% CI (–0.819, –0.262)], control [M_diff_ = –0.233, SE_diff_ = 0.061, *t*_(57.0)_ = −3.814, *p*_corrected_ = 0.004, 95% CI (–0.803, –0.248)], large [M_diff_ = –0.266, SE_diff_ = 0.060, *t*_(57.0)_ = −4.412, *p*_corrected_ ≤ 0.001, 95% CI (–0.884, –0.323)] and extra-large [M_diff_ = –0.249, SE_diff_ = 0.056, *t*_(57.0)_ = −4.454, *p*_corrected_ ≤ 0.001, 95% CI (–0.926, –0.345)]. Results are shown in Figure 4. Descriptives and significance values for physiological measures are presented in Extended Data Figures 4-1 and 4–2.

10.1523/ENEURO.0104-22.2022.f4-1 Extended Data Figure 4-1 Descriptives (mean/SD) for physiological analysis. Download Figure 4-1, DOCX file.

10.1523/ENEURO.0104-22.2022.f4-2 Extended Data Figure 4-2 Statistical significance values for physiological measures. Note: statistics are derived from one-way repeated measures ANOVAs. *p*, *p* value; FDR, FDR correction. Download Figure 4-2, DOCX file.

### No association between self-reported emotion and changes in physiological response

It is important to understand whether participants can accurately identify changes to their emotional state. Currently, accepted practice during postoccupancy evaluations of buildings is to complete surveys with building users to understand whether their needs are being met. However, the degree to which subjective emotional judgments are associated with electrophysiological measures related to emotion is unclear. During the experiment, participants provided self-reports of their emotional state using the Self-Assessment Manikin. Self-report of pleasure showed an effect of condition (*F*_(4,236)_ = 12.0, *p* ≤ 0.001, *η*^2^ = 0.156). *Post hoc* comparisons showed significant positive increases between resting state and all conditions, small [M_diff_ = 0.727, SE_diff_ = 0.123, *t*_(65.0)_ = 5.904, *p*_corrected_ ≤ 0.001, 95% CI (–0.819, –0.262)], control [M_diff_ = 0.591, SE_diff_ = 0.126, *t*_(65.0)_ = 4.695, *p*_corrected_ ≤ 0.001, 95% CI (–0.803, –0.248)], large [M_diff_ = 0.576, SE_diff_ = 0.122, *t*_(65.0)_ = 4.709, *p*_corrected_ ≤ 0.001, 95% CI (–0.884, –0.323)], and extra-large [M_diff_ = 0.591, SE_diff_ = 0.126, *t*_(65.0)_ = 4.695, *p*_corrected_ ≤ 0.001, 95% CI (–0.926, –0.345)], but did not reveal significant differences between scale conditions. No significant effects were observed for self-reports of arousal (*F*_(4,245)_ = 1.36, *p *=* *0.251, *η*^2^ = 0.020) or dominance (*F*_(3,225)_ = 1.78, *p *=* *0.143, *η*^2^ = 0.027).

**Figure 5.**
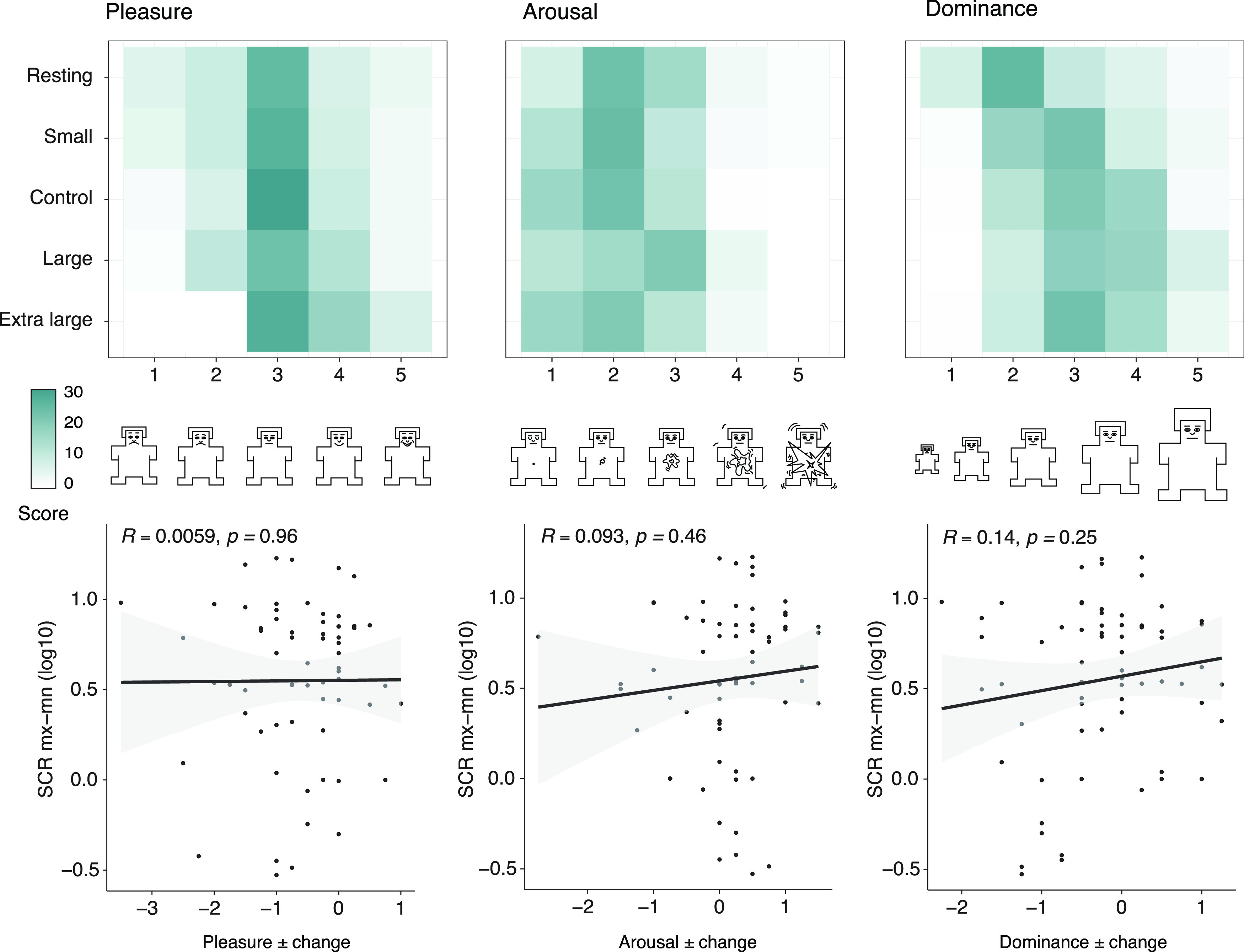
Correlations between self-assessment and physiological response. (Top row) Heatmap of the aggregated self-report responses across participants for pleasure, arousal, and dominance. (Middle row) The pictorial scale on the x-axis depicts the SAM dimensions of pleasure, arousal, and dominance. (Bottom row) Correlations were used to understand if a relationship between physiological response and self-report could be found for pleasure, arousal, and dominance. The data was obtained from averaging the response to built environment conditions and obtaining the absolute difference to the resting state condition for SCR Mx-Mn and the ± value from each domain in the self-assessment manikin.

Using the baseline resting state scores as a comparator, we analyzed whether participants rated themselves higher or lower for each of the three measures and compared this to the direction of change in the most responsive physiological measure, SCR Mx-Mn. Using Pearson’s *r* correlations, we found no relationship between the direction of SCR Mx-Mn change and self-report change across the three dimensions of pleasure (*r* = 0.006, *p *=* *0.96), arousal (*r* = 0.093, *p *=* *0.46), and dominance (*r* = 0.14, *p *=* *0.025) shown in Figure 5.

### We did not detect a relationship between potential confounding variables such as order of exposure and IEQ range with the neurophysiological results

As each participant experienced the resting state before a randomized set of conditions, we checked for stimulus habituation by comparing results of SCR Mx-Mn, and the averaged γ EEG power spectra density with the order of exposure presented to each participant. We did not find a positive or negative linear relationship, which argues against the possibility that the difference between resting state and the scale conditions was because of the exposure order.

We measured IEQ variables within the CAVE throughout the study. Air temperature (°C) was stratified across three height levels of low (M = 22.2 ± SEM = 0.108), mid (M = 22.2 ± SEM = 0.106), and high (M = 22.3 ± SEM = 0.105). Wet-bulb globe temperature (°C), which measures apparent temperature, was stratified across four height levels of low (M = 22.1 ± SEM = 0.106), mid (M = 22.2 ± SEM = 0.102), high (M = 22.2 ± SEM = 0.104), and approximate head height for standing position (M = 23.1 ± SEM = 0.111). Air velocity (m/s) was also stratified across four levels of low (M = 0.076 ± SEM = 0.003), mid (M = 0.069 ± SEM ≤ 0.001), high (M = 0.070 ± SEM = 0.001), and head (M = 0.006 ± SEM ≤ 0.001). We also recorded overall relative humidity (%; M = 45.4 ± SEM = 0.781) and carbon dioxide in parts per million (ppm; M = 572 ±SEM = 2.92). Noise levels (dB) fluctuated because of mechanical projector lamp ventilation (M = 46.7 ± SEM = 0.383). Results are shown in Extended Data Figure 2-1.

### We did not detect a relationship between personality and autonomic responsiveness to conditions

To understand whether personality played a role in response to the built environment, we tested whether differences in participants’ personality accounted for differences in response to the built environment. Participants completed the abbreviated International Personality Inventory Pool (IPIP-NEO-120) before the experiment. To check for an association between autonomic reactivity in the built environment and personality we ran a correlation analysis using the most reactive physiological measure, SCR Mx-Mn. We did not observe any correlations as presented in Figure 7.

## Discussion

**Figure 6.**
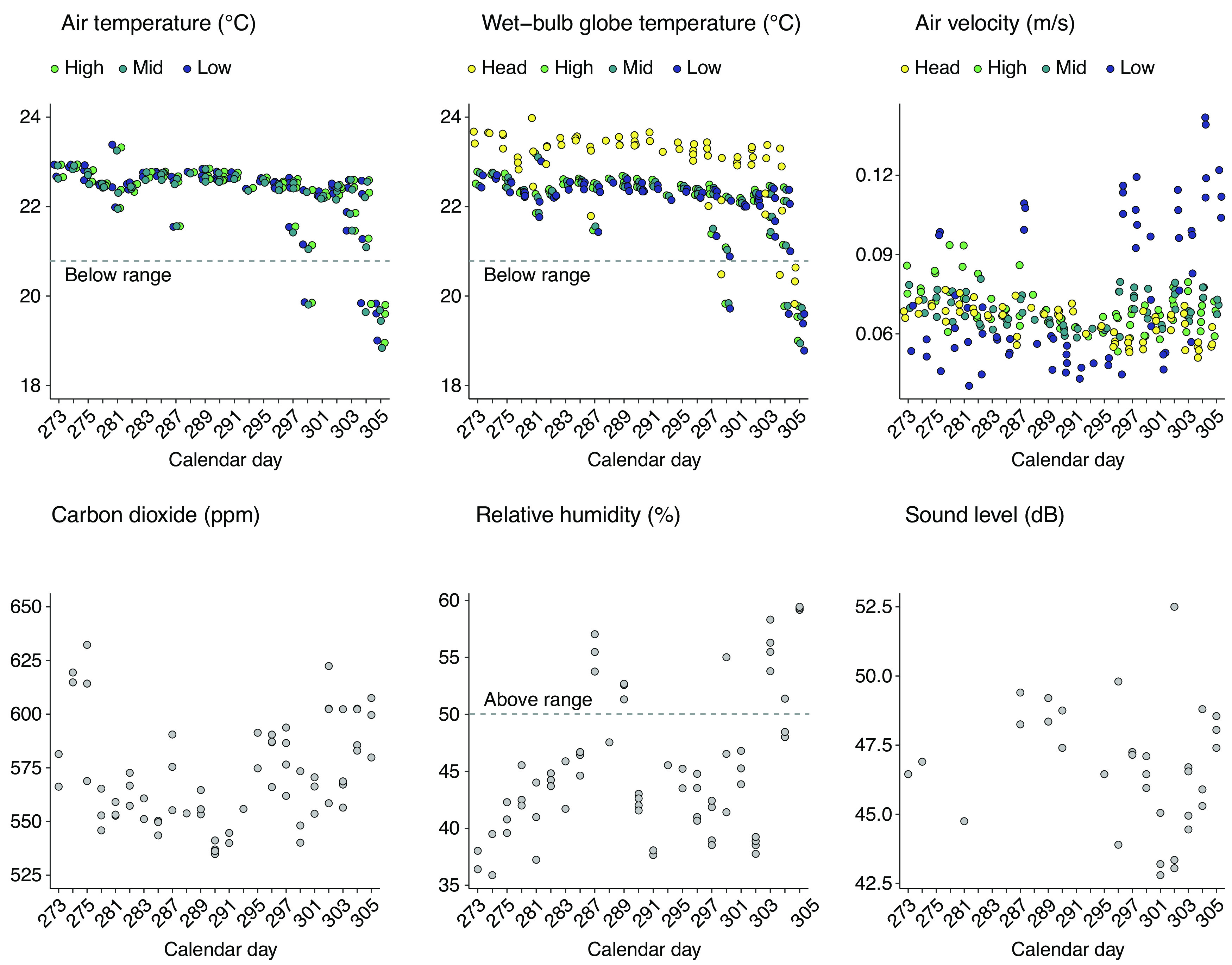
Exposure order and comfort conditions. All IEQ data are organized by calendar day the reading was collected on (*x*-axis). Multiple points represent the number of participants from each day. Measurements stratified by height data were required to accommodate differences in temperature and air velocity.

With limited exploratory work conducted in the field (Bower, 2019), this study is the first to test how the scale of the built environment affects emotional and neurophysiological response with a rigorously controlled method, using VR and IEQ monitoring. This study demonstrates that enlarged scale had a significant impact on brain oscillatory activity in the β, low-γ and high-γ bandwidths, even after controlling for potentially confounding variables such as stimulus habituation (Tang et al., 2018) and thermal comfort (see Fig. 6). We also detected increases in measures of range for skin conductance (Mx-Mn slope) and HRV (root mean square of successive differences) to the built environment conditions, but not scale. Scale of the built environment was not seen to modulate autonomic response or anticipated EEG measures of frontal midline power or frontal lateralization within the θ and α bandwidths across participants. However, during a posteriori analysis we found increased frontal midline power in the low-γ and high-γ bandwidth, associated with increased scale between control to extra-large, and large to extra-large conditions. We also found increased left lateralization in the γ bandwidth between the large and extra-large condition, suggesting changes in γ midline power and lateralization may be a physiological marker of the impact of built environment scale. The study confirmed our hypothesis that participants’ self-report of emotion for the dimensions of arousal and dominance do not correspond with autonomic or brain wave modulations. We did find a significant difference in self-report of pleasure between resting state and conditions, but not between scales, and this difference was not seen in self-report of arousal or dominance.

**Figure 7.**
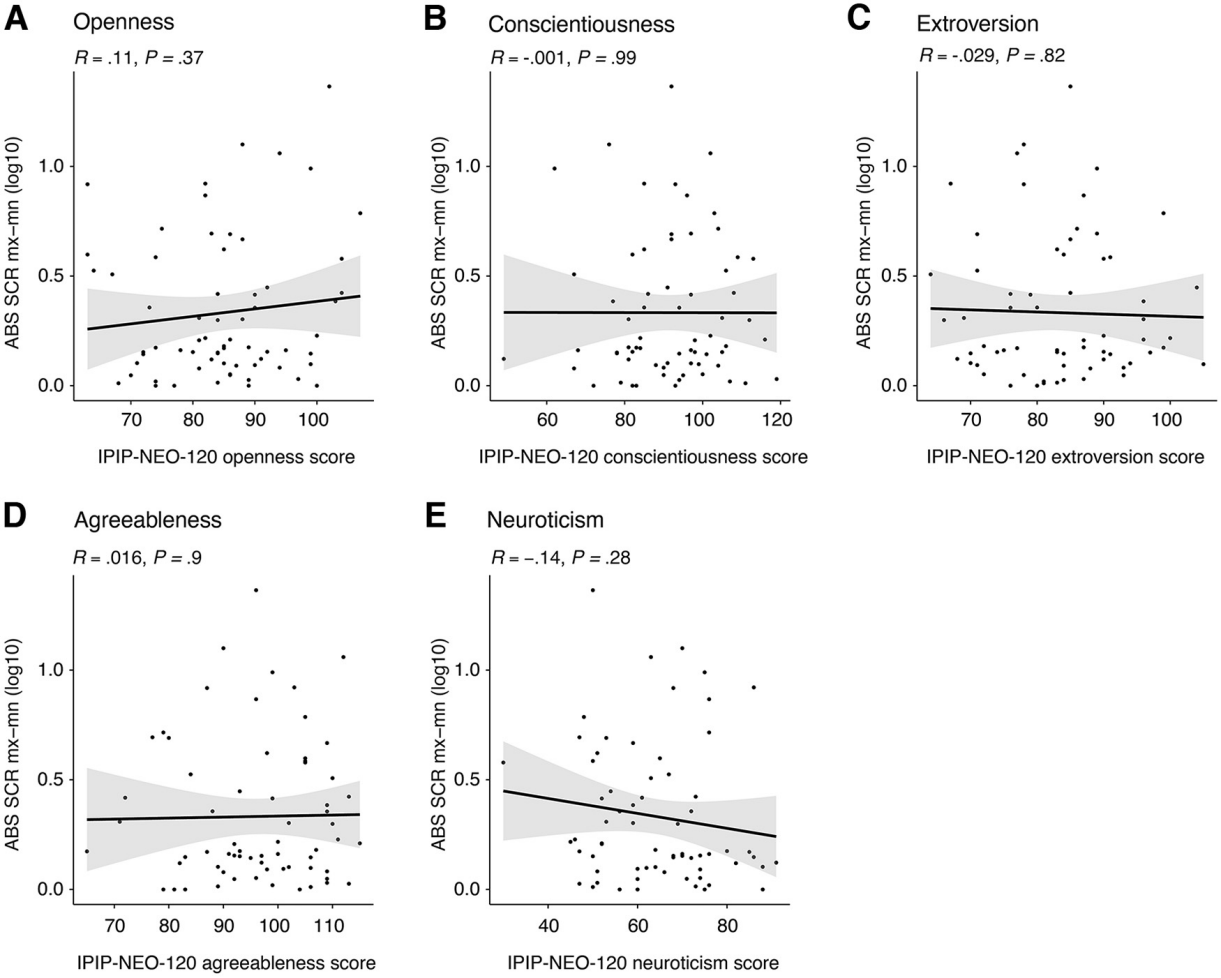
Exploratory correlations between personality measures and physiological response. ***A–E***, Correlations between Openness, Conscientiousness, Extroversion, Agreeableness and Neuroticism (OCEAN) big five personality traits and the absolute difference in the averaged response across built environment conditions for skin conductance response Mx-Mn slope value (SCR Mx-Mn). No correlation was found.

The results of this study indicate that changes in HRV and SCR do occur during built environment exposures, which are modulated through the autonomic nervous system. This has been thought to correspond with limbic system activation, which is involved in our behavioral and emotional response. It is important to distinguish that these lowered levels of HRV and elevated SCR to the built environment scenes do not equate to a positive emotion or a better environment for our health. Likewise, we cannot rule out that these changes will result in long-term negative effects, however research shows elevated arousal and stress over a long period of time can be detrimental to our health (Schneiderman et al., 2005). Instead, this research provides the first step in demonstrating that the presentation of a virtual built environment, compared with resting state, modulates autonomic activity in measures of sympathetic and parasympathetic activity.

There are multiple theories for hemispheric lateralization in emotional processing studies. Increased asymmetry of the right hemisphere has been associated with emotional stimuli, regardless of valence (Müller et al., 1999, 2000). From a neurocognitive perspective, it is unclear whether this relates to emotion processing, or other attentional or perceptual processes related to an enlarged built environment scale. We also detected EEG power spectra in the β bandwidth increased from the small to the extra-large, control to the extra-large, and the large to the extra-large but did not differ between scale conditions for the remaining bandwidths. However, we did find significant differences between resting state and the scale conditions across most bandwidths. In contrast to our hypothesis, we did not detect increased α and θ frontal midline power or lateralization across scale conditions, which is associated with positive emotional response (Davidson, 1992; Ekman and Davidson, 1993). These findings suggest that although scale may not be involved in emotional processing, it may influence high-frequency oscillatory processes, such as working memory and decision-making (Spitzer and Haegens, 2017). However, we acknowledge that without source localization we are inferring the neural activity, and therefore our interpretations of the effects remain speculative. Future research exploring higher-frequency signals with EEG could consider using an analysis approach incorporating source localization of the EEG to aid reducing the impact of any eye movement artifact in the signal (Carl et al., 2012; Hipp and Siegel, 2013). Another option for further research could be to use a data driven approach using large samples to perform quantitative EEG analysis.

The study also revealed that self-report of emotion was not an accurate indicator for increased autonomic nervous system response. Emotion processing studies investigating alignment between self-report and physiological indicators remain inconsistent. With some studies reporting consistency (Hagemann et al., 1998), while others remain inconsistent (Kassam and Mendes, 2013). Despite the lack of current consensus, this is an important finding for design professionals, as it indicates the need to shift practice in postoccupancy evaluation of buildings. We suggest the findings highlight the need to go beyond self-report and observational data alone, as these do not capture effects that may not be consciously perceived or comprehended.

There is evidence widespread high-frequency activity is increased during a range of complex cognitive tasks (Simos et al., 2002; Fitzgibbon et al., 2004). As we found preliminary evidence for the effect of enlarged scale in the higher frequency bandwidths, future studies are warranted to integrate this further. This could include a working memory activity during exposure to the built environment conditions, which could clarify whether task-based performance is impacted (Jensen et al., 2007). Previous studies have shown that during tasks where participants are required to perform a range of cognitive tasks to induce stress, indoor environmental factors such as temperature (Silva et al., 2019) and view to nature (Fich et al., 2014) modulate physiological response and impact performance. As it is suggested we have a threshold of tolerance to stressors, modulated by gene-environment interactions (Caspi and Moffitt, 2006), the built environment could act to increase or reduce the tolerance. Therefore, exposing participants to higher stress may heighten the effect of the built environment on neurophysiological response. It may also be that examining network-level responses through a technique, such as functional connectivity, is required to understand whether scale has an effect on neural activity. Studies have indicated that techniques with greater temporal resolution may be more effective for detecting brain activity when measuring for emotional state change (Bekkedal et al., 2011).

As the study is exploratory, further work understanding the interplay between design elements is required. It is expected that this technique and singular approach can be further used with different design elements with larger, more complex scenes. The scene created was purposefully designed to be context neutral. This meant it was devoid of color, materiality/excessive texture, atypical geometry, and furnishings, which may indicate to the participant the context/setting. However, this is not realistic as we do not experience environments that have so little visual information. This study also relied on visual information processing to understand the effect of scale. Work is required to understand whether similar physiological activity and neural encoding occurs when processing the built environment through other sensory modalities such as the auditory system through processing reverberation feedback to determine the scale of the space. Future research could steadily progress in complexity by exploring how these design elements of the environment interact with other enrichment components through studies involving motor activity, cognitive stimulation, and the presence of other people in the space.

The study also limited participants to those self-reporting they had no underlying mental health conditions. This may mean a broader more inclusive sample will enable us to understand whether the built environment impacts those with preexisting psychological, psychiatric, neurodevelopmental, and neurodegenerative conditions to a greater extent than the study sample.

Active debate continues over the ecological validity of virtual environments to simulate physical environments (Sanchez-Vives and Slater, 2005; Kalantari et al., 2021). VR enables a high level of environmental control over the design, testing and is cost-efficient when compared with the construction of physically built environments. While studies have explored the difference between virtually experienced and physically experienced spaces, in this research, it was found that a CAVE can be a cost-effective method for the development of a controlled environment. Future work replicating the approach in physically created scaled spaces would be beneficial to understand whether differences in responses to the two modalities exist.

The ability for built environment design to modulate neural processing may have implications on our cognitive, attentional, perceptual, and emotional functioning. With the potential to deliver significant public health, economic and social benefits to the entire community. This work generates new knowledge for industry and policy makers to enable enhanced understanding, prediction, and optimization of built environment design. It is important that attention is drawn to pursuing future studies that investigate whether built environment design can provide a neuroprotective factor for individuals who are at increased risk of developing a psychological disorder because of other environmental and epigenetic stressors. This study provides a rigorous empirical framework for assessing the impact of the built environment on human emotion for future studies. The findings confirm that the buildings we inhabit play a role in determining our health.

## Synthesis

Reviewing Editor: Niko Busch, Westfalische Wilhelms-Universitat Munster Decisions are customarily a result of the Reviewing Editor and the peer reviewers coming together and discussing their recommendations until a consensus is reached. When revisions are invited, a fact-based synthesis statement explaining their decision and outlining what is needed to prepare a revision will be listed below. The following reviewer(s) agreed to reveal their identity: Oliver Baumann, Christoph Michel.

Thank you for submitting the revised version of your manuscript. The reviewers and I have evaluated your manuscript once more, and I am happy to let you know that we have no further comments or requests for revisions. Congratulations!

Kind regards,

Niko Busch

## Author Response

Responses for revision

Review Synthesis

1) While I appreciate the straightforward distinction between a priori and exploratory hypotheses, I agree with the reviewers that even exploratory hypotheses require some degree of justification and contextualization. This is also related to reviewer 2, point 3 about the selection of EEG channels for an exploratory analysis of high-frequency activity, which was based on literature about an entirely different frequency band.

Thank you for identifying this gap. We have added and/or point to the following text into the Introduction and Discussion to justify and contextualize investigating higher frequency bandwidths.

Introduction: Page 5, Lines 94-109 “As this field of research is early in development, in conjunction with our a priori hypotheses, we opted to perform exploratory analyses across the remaining EEG power spectra regions of interest (ROIs) and of the overall power spectral density. This approach was selected as we were interested in understanding whether the built environment may affect other cognitive functions such as perception, attention, and memory which have been associated with higher frequency oscillatory activity. Neural oscillations in the gamma frequency range have been associated with visual tasks such as perception (Keil, Müller, Ray, Gruber, & Elbert, 1999), attention (Müller, Gruber, & Keil, 2000), and memory (Tallon-Baudry, Bertrand, Peronnet, & Pernier, 1998). We expected changes to scale would result in increased frontal midline power and frontal hemispheric lateralization in the theta and alpha bandwidths, due to their association with emotion (Aftanas & Golocheikine, 2001; Coan & Allen, 2004; Davidson, 2004). We also hypothesized scale conditions would increase baseline autonomic measures, and self-report may not reflect underlying autonomic or EEG modulations.”

Discussion: Page 32-33, Lines 641-646 “In contrast to our hypothesis, we did not detect increased alpha and theta frontal midline power or lateralization across scale conditions, which is associated with positive emotional response (Davidson, 1992; Ekman & Davidson, 1993). These findings suggest that although scale may not be involved in emotional processing, it may influence high frequency oscillatory processes, such as working memory and decision making (Spitzer & Haegens, 2017).”

Discussion: Page 33-34, Lines 665-671 “There is evidence widespread high-frequency activity is increased during a range of complex cognitive tasks (Fitzgibbon, Pope, Mackenzie, Clark, & Willoughby, 2004; Simos, Papanikolaou, Sakkalis, & Sifis, 2002). As we found preliminary evidence for the effect of enlarged scale in the higher frequency bandwidths, future studies are warranted to integrate this further. This could include a working memory activity during exposure to the built environment conditions, which could clarify if task-based performance is impacted (Jensen, Kaiser, & Lachaux, 2007).

2) I second reviewer 2’s skepticism regarding the EEG analysis, and I strongly encourage you to consider his advice. I would also like to emphasize his concerns regarding the possible contamination of the EEG data by eye movements. Specifically, high-frequency activity in scalp-recorded EEG has long been discredited as a possible signal-processing artifact due to miniature eye movements (Yuval-Greenberg, S., Tomer, O., Keren, A. S., Nelken, I., & Deouell, L. Y. (2008). Transient induced gamma-band response in EEG as a manifestation of miniature saccades. Neuron, 58(3), 429-441.). Given that the findings in the gamma band are among the central results of this study, it is essential to verify that they are not due to eye movement artifacts.

Thank you for highlighting this study, it’s a great area of research to explore in future studies. While we acknowledge that investigating high-frequency signals with EEG is a limitation of our study design, we don’t believe the results are contaminated from miniature eye movement artifact and have taken steps to try and minimise the possibility of these artifacts in our data. Here we’ve listed a few reasons why we don’t believe our results are due to eye movement artifacts.

• We used a multi-step preprocessing approach to aid in artifact removal. This included the use of independent component analysis (ICA) incorporating the ICLabel classifier (Pion-Tonachini, Kreutz-Delgado, & Makeig, 2019) to isolate and remove various artifactual components (e.g., muscle, eye, heart, line noise, channel noise). The ICLabel classifier was developed using an extensive training set (6,352 individual EEG recordings), and has been validated against many other publicly available classifiers, the results of which indicate that it performs either better than, or comparably to these other classifiers. In addition, we also further utilized EEGLab functions to remove bad channels (via EEGLAB’s ’pop_rejchan’ function) and any remaining noisy epochs (via the ’pop_eegthresh’ function). Finally, we also incorporated the source-estimate-utilizing noise-discarding algorithm (SOUND) (Mutanen, Metsomaa, Liljander, & Ilmoniemi, 2018) - a noise cleaning method that has been shown to offer robust removal of noise in the EEG signal using Wiener estimators. We believe that this comprehensive preprocessing pipeline will have aided in greatly reducing the presence of artifact within the EEG signal.

References:

Pion-Tonachini, L., Kreutz-Delgado, K., & Makeig, S. (2019). ICLabel: An automated electroencephalographic independent component classifier, dataset, and website. NeuroImage, 198, 181-197. https://www.doi.org/10.1016/j.neuroimage.2019.05.026

Mutanen, T. P., Metsomaa, J., Liljander, S., & Ilmoniemi, R. J. (2018). Automatic and robust noise suppression in EEG and MEG: The SOUND algorithm. NeuroImage, 166, 135-151. https://www.doi.org/10.1016/j.neuroimage.2017.10.021

Methods: Pages 15-16, Lines 319-347 “The EEG data were preprocessed using EEGLab (v2019.1) (Delorme & Makeig, 2004), an open-source graphic user interface and toolbox plugin for MATLAB R2019b (v9.7.0.1471314, MathWorks, Inc). We applied a bandpass filter from 1 to 70 Hz (zero-phase Butterworth filter) on continuous EEG data. A 47-53 Hz notch-filter was applied to exclude electrical interference from the CAVE environment. We then removed eye channels and the Cz reference channel. Next, we rejected channels if the kurtosis value was >5 standard deviations outside the average and replaced information in those channels using a spherical spline interpolation. Data were subsequently re-referenced to the average of all electrodes. To aid the removal of recording noise we applied the SOUND algorithm using input parameters of 5 iterations to evaluate noise in each channel and 0.2 regularization level (lambda value) to control the amount of cleaning (Mutanen, Metsomaa, Liljander, & Ilmoniemi, 2018). Each participant’s continuous EEG data were decomposed using independent component analysis (FastICA algorithm) (Hyvärinen & Oja, 2000), with artifactual components identified with assistance from the ICLabel plugin (Pion-Tonachini, Kreutz-Delgado, & Makeig, 2019). A component was removed if ICLabel classified the probability of that component containing brain data was less than 30% and the component was not in the ’other’ category. The mean of the components removed for each subject was 6.16 {plus minus} 3.92.

Using the time-stamped event markers in the continuous recording, each file was then split into 120 second block files using the start marker for each condition. Data were segmented into three-second epochs for subsequent analyses. Finally, additional artefact rejection was performed to remove any remaining noisy epochs with data exceeding {plus minus} 150 μV using the EEGLab ’pop_eegthresh’ function. After cleaning we calculated the average epochs remaining for each condition and participant (mean number of epochs = 39.5, {plus minus} 1.46). Lastly, we converted data from each participant/electrode to the frequency domain using the Fast Fourier Transform (FFT) with Hanning taper in the FieldTrip toolbox for EEG/MEG-analysis (1 Hz frequency steps between 1 to 70 Hz) (Oostenveld, Fries, Maris, & Schoffelen, 2011).

• Our data was obtained from eyes open resting-state recordings rather than an event-related potential (ERP) study paradigm. We note most papers indicating micro saccade-related artifacts are based on ERP studies, where activity is time-locked to the stimulus (Carl, Açık, König, Engel, & Hipp, 2012; Hipp & Siegel, 2013; Keren, Yuval-Greenberg, & Deouell, 2010). Our study instead measured response over a two-minute (120 second) period and then was segmented into three-second epochs and averaged power spectral density.

References:

Carl, C., Açık, A., König, P., Engel, A. K., & Hipp, J. F. (2012). The saccadic spike artifact in MEG. NeuroImage, 59(2), 1657-1667. https://www.doi.org/10.1016/j.neuroimage.2011.09.020

Hipp, J. F., & Siegel, M. (2013). Dissociating neuronal gamma-band activity from cranial and ocular muscle activity in EEG. Front Hum Neurosci, 7, 338. doi:10.3389/fnhum.2013.00338

Keren, A. S., Yuval-Greenberg, S., & Deouell, L. Y. (2010). Saccadic spike potentials in gamma-band EEG: characterization, detection and suppression. NeuroImage, 49(3), 2248-2263. https:///www.doi.org/10.1016/j.neuroimage.2009.10.057

Methods: Page 16, Lines 338-340 “Using the time-stamped event markers in the continuous recording, each file was then split into 120 second block files using the start marker for each condition. Data were segmented into three-second epochs for subsequent analyses.”

• We also went sought to minimise artifacts in our study design by ensuring the participants were comfortably seated with well fitted stereoscopic glasses (we fitted cotton wool under the nose bridge if the glasses showed any movement). We instructed participants to view the scene and their location in the CAVE allowed them a clear field of view which did not require head movement. We also note the researcher monitored the participant (and the recordings) throughout the experiment to ensure minimal movements occurred.

Methods: Pages 13-14, Lines 212-218 “Participants were seated for the duration of the experiment and instructed to pay attention to the scene they were presented. We elected to run a static resting state study where participants sat immersed in the space, rather than setting a task involving movement through thresholds, as this has been thought to affect cognition and memory (Pettijohn & Radvansky, 2016). By sitting, any height differences were also minimized between participants, and this also helped to minimize any movement-related artefacts in the electrophysiological measures.”

Methods: Page 9, Lines 184-187 “A pair of stereoscopic glasses to view the CAVE projection were then carefully fitted on top of the EEG cap. These remained on throughout the experiment. Impedances were checked and adjusted when necessary to ensure the quality of electrode-to-scalp contact.”

Methods: Page 12, Lines 245-249 “A wooden fixed chair with a seat pad for comfort and back support for posture consistency was positioned in the center of the CAVE, effectively within the center of each virtual room regardless of scale. The chair remained in the central position to ensure all participants were situated in the same location.”

• Next, despite the extensive steps we’ve taken to minimize artifacts in the data, we acknowledge there are limitations to investigating higher-frequency signals with EEG. For instance, future studies wanting to explore beta and gamma activity could consider utilizing an analysis approach incorporating source localization of the EEG signal, which might further aid in reducing the impact of any eye movement artifact on the signal (Carl, Açık, König, Engel, & Hipp, 2012; Hipp & Siegel, 2013). As individual MRI scans were not performed for the current study, this was not feasible in our case. Future studies might also consider incorporating additional concurrent video eye tracking. However, this would be likely to increase the complexity (and cost) of the study, and could introduce potential technical difficulties (Keren, Yuval-Greenberg, & Deouell, 2010).

Keren, A. S., Yuval-Greenberg, S., & Deouell, L. Y. (2010). Saccadic spike potentials in gamma-band EEG: characterization, detection and suppression. NeuroImage, 49(3), 2248-2263. https://www.doi.org/10.1016/j.neuroimage.2009.10.057

Carl, C., Açık, A., König, P., Engel, A. K., & Hipp, J. F. (2012). The saccadic spike artifact in MEG. NeuroImage, 59(2), 1657-1667. https://www.doi.org/10.1016/j.neuroimage.2011.09.020

We have added a statement on this to our Discussion:

Discussion: Page 33, Lines 646-652 “However, we acknowledge that without source localization we are inferring the neural activity, and therefore our interpretations of the effects remain speculative. Future research exploring higher-frequency signals with EEG could consider utilizing an analysis approach incorporating source localization of the EEG to aid reducing the impact of any eye movement artifact in the signal (Carl, Açık, König, Engel, & Hipp, 2012; Hipp & Siegel, 2013).”

3) The study includes an analysis of a large number of physiological parameters. You report that “To control for multiple comparisons, a false discovery rate (FDR) 328 correction was applied to the results”. I would appreciate if you could unpack that a bit. Specifically, the EEG analysis was conducted for several frequency bands and regions of interest. How were results corrected for multiple comparisons in that case?

We used the False Discovery Rate (FDR) correction here to account for multiple comparisons both across conditions and across frequency bands. We also note the number following “false discovery rate (FDR) 328” is a referencing mistake - thank you for spotting this, we have fixed it as below. We have also added an additional reference explaining and validating this technique and have provided the full citation reference below.

Materials and Methods, Data analysis: Page 17, Lines 367-371 “To correct for multiple comparisons where significance was detected within-subjects, the false discovery rate (FDR) method was used (Benjamini & Hochberg, 1995). The FDR is an alternative approach to multiple testing which increases detection power over traditional methods for multiple testing (Genovese, 2015).”

• Benjamini, Y., & Hochberg, Y. (1995). Controlling the False Discovery Rate: A Practical and Powerful Approach to Multiple Testing. Journal of the Royal Statistical Society. Series B (Methodological), 57(1), 289-300. https://www.doi.org/10.1111/j.2517-6161.1995.tb02031.x

• Genovese, C. R. (2015). False Discovery Rate Control. In A. W. Toga (Ed.), Brain Mapping (pp. 501-507). Waltham: Academic Press.

Reviewer 1

1) My only recommendations concern the hypotheses. Firstly, while I commend the authors that they are straightforward about the clear distinction of their a priori and exploratory hypotheses, more detail regarding the exploratory hypotheses will nevertheless be helpful. i.e., Why were the high-frequency oscillations of interest, and to which cognitive and perceptual processes would they relate (including relevant references)? While this is brought up in the discussion, an earlier description would be helpful. Secondly, more detail would be beneficial regarding why the self-reports were predicted to not align with the electrophysiological measures.

We have added an earlier description of the high-frequency oscillations, relevant references, and why we analysed all the data rather for completeness.

Introduction: Page 5, Lines 94-109 “As this field of research is early in development, in conjunction with our a priori hypotheses, we opted to perform exploratory analyses across the remaining EEG power spectra regions of interest (ROIs) and of the overall power spectral density. This approach was selected as we were interested in understanding whether the built environment may affect other cognitive functions such as perception, attention, and memory which have been associated with higher frequency oscillatory activity. Neural oscillations in the gamma frequency range have been associated with visual tasks such as perception (Keil, Müller, Ray, Gruber, & Elbert, 1999), attention (Müller, Gruber, & Keil, 2000), and memory (Tallon-Baudry, Bertrand, Peronnet, & Pernier, 1998). We expected changes to scale would result in increased frontal midline power and frontal hemispheric lateralization in the theta and alpha bandwidths, due to their association with emotion (Aftanas & Golocheikine, 2001; Coan & Allen, 2004; Davidson, 2004). We also hypothesized scale conditions would increase baseline autonomic measures, and self-report may not reflect underlying autonomic or EEG modulations.”

We expected self-report not to align with electrophysiological measures because research has questioned whether people have difficulty understanding their emotions, hesitate to give honest and/or undesirable responses and are subject to rationalizing their responses.

Discussion: Page 33, Lines 655-662 “The study also revealed that self-report of emotion was not an accurate indicator for increased autonomic nervous system response. Emotion processing studies investigating alignment between self-report and physiological indicators remain inconsistent. With some studies reporting consistency (Hagemann, Naumann, Becker, Maier, & Bartussek, 1998), while others remain inconsistent (Kassam & Mendes, 2013). Despite the lack of current consensus, this is an important finding for design professionals, as it indicates the need to shift practice in post occupancy evaluation of buildings.”

-

Reviewer 2

The EEG analysis requires some clarification:

1) Effects were found in the Beta and Gamma band which are most prone to movement artifacts. While ICA was applied, I wonder whether this was sufficient to eliminate head and eye movement artifacts that most probably increased from baseline (rest) to the room condition and with increasing scale of the room. It is important to report about the number and the topography of the ICA components that were removed in each of the conditions. Also: how was head movement controlled for? This seems to me more important than assuring air temperature, velocity, and humidity.

Thank you for recognizing we’ve applied ICA. We have reported the number and topography of the ICA components removed and provided this data on open science framework with our other materials, alongside an example of ICA decomposition and the components removed. We have also created an overall average and inserted this into the methods and pointed to the supplementary data. We also want to highlight our approach was multi-pronged to deal with artifact in the signal. This included the removal of bad trials and channels using EEGLab functions, as well as using the SOUND algorithm, which includes spatial Wiener filtering to remove noise from the signal.

Methods: Pages 15-16, Lines 319-347 “The EEG data were preprocessed using EEGLab (v2019.1) (Delorme & Makeig, 2004), an open source graphic user interface and toolbox plugin for MATLAB R2019b (v9.7.0.1471314, MathWorks, Inc). We applied a bandpass filter from 1 to 70 Hz (zero-phase Butterworth filter) on continuous EEG data. A 47-53 Hz notch-filter was applied to exclude electrical interference from the CAVE environment. We then removed eye channels and the Cz reference channel. Next, we rejected channels if the kurtosis value was >5 standard deviations outside the average and replaced information in those channels using a spherical spline interpolation. Data were subsequently re-referenced to the average of all electrodes. To aid the removal of recording noise we applied the SOUND algorithm using input parameters of 5 iterations to evaluate noise in each channel and 0.2 regularization level (lambda value) to control the amount of cleaning (Mutanen, Metsomaa, Liljander, & Ilmoniemi, 2018). Each participant’s continuous EEG data were decomposed using independent component analysis (FastICA algorithm) (Hyvärinen & Oja, 2000), with artifactual components identified with assistance from the ICLabel plugin (Pion-Tonachini, Kreutz-Delgado, & Makeig, 2019). A component was removed if ICLabel classified the probability of that component containing brain data was less than 30% and the component was not in the ’other’ category. The mean of the components removed for each subject was 6.16 {plus minus} 3.92.

Using the time-stamped event markers in the continuous recording, each file was then split into 120 second block files using the start marker for each condition. Data were segmented into three-second epochs for subsequent analyses. Finally, additional artefact rejection was performed to remove any remaining noisy epochs with data exceeding {plus minus} 150 μV using the EEGLab ’pop_eegthresh’ function. After cleaning we calculated the average epochs remaining for each condition and participant (mean number of epochs = 39.5, {plus minus} 1.46). Lastly, we converted data from each participant/electrode to the frequency domain using the Fast Fourier Transform (FFT) with Hanning taper in the FieldTrip toolbox for EEG/MEG-analysis (1 Hz frequency steps between 1 to 70 Hz) (Oostenveld, Fries, Maris, & Schoffelen, 2011).

We recognize and have tried to address concerns regarding high-frequency activity in the signal given the overlap with low signal-to-noise-ratio and overlap with muscle spiking. Given the strong overlap in frequency range between the EMG activity and beta/gamma oscillations

Head movement was minimized through the seated posture of the participant and the clear field of view which did not require large head movement as the door, chair, and wall they were facing were all in view. As also mentioned in a comment above, we note the researcher monitored the participant (and the recordings) throughout the experiment to ensure minimal movements occurred.

Methods: Page 9, Lines 192-194 “By sitting, any height differences were also minimized between participants, and this also helped to minimize any movement-related artefacts in the electrophysiological measures.”

We note the indoor air quality (IAQ) variables were recorded for physiological purposes (rather than us expecting the IAQ to affect ambient air movement interfering with the electrodes). It is important to report physiological indicators as a potential confounding variable to neurophysiological processes. The following text explains this:

Methods: Page 10, Lines 201-206 “We measured indoor environmental quality (IEQ) variables within the CAVE throughout the study. These measures were analysed to ensure any fluctuations to these properties linked to data which may influence emotion and neurophysiological response were minimized. To reduce the chance of negative influence all data were collected over a consecutive period in spring to reduce heat/cool load on the building triggering changes in the heating, ventilation, and air conditioning system.”

2) The power maps in Figure 2 are suspicious: why is the power minimal in the electrodes around Cz? It is said that the reference electrode Cz was removed and the average reference was calculated. Why was Cz removed? The reference electrode should be included when calculating the average reference. It would be helpful to show the power maps of the other frequency bands, particularly of the Alpha band and show that these maps have the typical power distribution (e.g. occipital Alpha).

The EEG system we used (EGI GSN Hydrocel 64 channel cap) has the Cz electrode as the set reference channel. We have explicitly stated in the manuscript that: 1) The Cz was not included in our analyses and 2) We have used a prioiri electrode regions of interest (ROIs), so even if we were to include the Cz these results would not be altered (as none included the Cz electrode). We therefore don’t feel we are misleading readers regarding the electrodes we have selected to analyse. However, to provide greater coverage across the vertex for visualization via the topographic plots, we have now replotted them after re-interpolating the Cz electrode back into the data. This is for visualization purposes only, which we have articulated within the legend of these figures and in the Method.

Methods: Page 9, Lines 173-176 “Data were acquired at a sampling rate of 1000 Hz, with Cz as the online reference. The Cz electrode was not included in our analysis, however, for the purposes of visualization, we have interpolated the Cz site for figures.”

We have also included a new supplementary figure of the remaining frequency bands (delta, theta and alpha) to show their power distribution. This shows the posterior dominant power in the alpha band.

Results: Pages 21, Line 446 Additional bandwidths are also presented in Extended Data Figure 2-3.

Figure Legends: Page 38, Lines 750-753 Figure 2-3: EEG topographies and boxplots with quartile ranges and medians for the overall power spectra in the delta, theta, and alpha bandwidths. Note the Cz site has been interpolated for this figure. (A) Delta 1 to 3 Hz; (B) Theta 4 to 7 Hz with amplitude range; and (C) Alpha 8 to 12 Hz.

Results: Page 21, Line 445 Results are shown in Figure 2.

Figure Legends: Page 37, Lines 738-744 Figure 2. Significant differences between the control and extra-large condition for EEG power spectral density was found in the beta bandwidth. To illustrate the differences, we have plotted EEG topographies and boxplots with quartile ranges and medians for the overall power spectra in the beta, low-gamma, and high-gamma bandwidths. Note the Cz site has been interpolated for this figure. (A) Beta 13 to 29 Hz; (B) Low-gamma 30 to 45 Hz with amplitude range; and (C) High gamma 55 to 70 Hz.

We also note and found additional papers which show similar topoplots using the same EGI EEG system with the Cz as the reference and showing distinctly lower power over the vertex (e.g., around the Cz site).

Neuhaus, E., Lowry, S.J., Santhosh, M. et al. Resting state EEG in youth with ASD: age, sex, and relation to phenotype. J Neurodevelop Disord 13, 33 (2021). https://doi.org/10.1186/s11689-021-09390-1

Levin, A.R., Varcin, K.J., O’Leary, H.M. et al. EEG power at 3 months in infants at high familial risk for autism. J Neurodevelop Disord 9, 34 (2017). https://doi.org/10.1186/s11689-017-9214-9

3) The analysis was restricted to frontal electrodes and to left-right hemispheric differences of frontal electrodes. It is argued that this selection was hypothesis driven with respect to literature showing frontal asymmetries related to emotion. However, this literature was reporting effects in the theta and alpha band, while the effects reported here are in the beta and gamma band. I recommend performing qEEG analysis of the power spectra using all electrodes and show the topographic differences.

We have added the remaining topoplots (delta, theta and alpha) as a Supplementary file to ensure the reader has access to the file results. This ensures all channels, and all bandwidths are reported on and available.

As this was an exploratory investigation, we were primarily interested in emotion processing, but we further wanted to explore regions of interest. We do note this is speculative. Quantitative EEG would be a significant deviation from our overall study design and would have required a larger sample to account for increased multiple comparisons (i.e., between electrodes). However, this is a valid point and we have addressed in the Discussion that future approaches could look at larger samples using data-driven approaches.

Introduction: Page 5, Lines 94-109 “As this field of research is early in development, in conjunction with our a priori hypotheses, we opted to perform exploratory analyses across the remaining EEG power spectra regions of interest (ROIs) and of the overall power spectral density. This approach was selected as we were interested in understanding whether the built environment may affect other cognitive functions such as perception, attention, and memory which have been associated with higher frequency oscillatory activity. Neural oscillations in the gamma frequency range have been associated with visual tasks such as perception (Keil, Müller, Ray, Gruber, & Elbert, 1999), attention (Müller, Gruber, & Keil, 2000), and memory (Tallon-Baudry, Bertrand, Peronnet, & Pernier, 1998). We expected changes to scale would result in increased frontal midline power and frontal hemispheric lateralization in the theta and alpha bandwidths, due to their association with emotion (Aftanas & Golocheikine, 2001; Coan & Allen, 2004; Davidson, 2004). We also hypothesized scale conditions would increase baseline autonomic measures, and self-report may not reflect underlying autonomic or EEG modulations.”

Discussion: Page 33, Lines 652-654 “Another option for further research could be to use a data driven approach using larger samples to perform quantitative EEG analysis.”

4) is well known that due to volume conduction, EEG scalp electrodes do not only record neuronal activity underlying them. It depends on the dipole orientation. Interpretations about the generators of the observed effects and about lateralization are purely speculative as long as no source localization is applied.

We acknowledge that without source localization approaches incorporating anatomical information derived from individual structural MRI scans, the results are speculative in relation to the precise neural generators of the signal recorded from the scalp. We have made sure this is now clearly articulated in the discussion, noting that future research can benefit from source localization through performing structural MRI scans in the study designs so source localization can be applied.

Discussion: Page 33, Lines 646-654 “However, we acknowledge that without source localization we are inferring the neural activity, and therefore our interpretations of the effects remain speculative. Future research exploring higher-frequency signals with EEG could consider utilizing an analysis approach incorporating source localization of the EEG to aid reducing the impact of any eye movement artifact in the signal (Carl, Açık, König, Engel, & Hipp, 2012; Hipp & Siegel, 2013).”

## References

[B1] Aftanas LI, Golocheikine SA (2001) Human anterior and frontal midline theta and lower alpha reflect emotionally positive state and internalized attention: high-resolution EEG investigation of meditation. Neurosci Lett 310:57–60. 10.1016/S0304-3940(01)02094-8 11524157

[B2] Ahern GL, Schwartz GE (1985) Differential lateralization for positive and negative emotion in the human brain: EEG spectral analysis. Neuropsychologia 23:745–755. 10.1016/0028-3932(85)90081-8 4080136

[B3] Alexander C, Ishikawa S, Silverstein M (1977) A pattern language: towns, buildings, construction. Oxford: Oxford University Press.

[B4] Barker TH, George RP, Howarth GS, Whittaker AL (2017) Assessment of housing density, space allocation and social hierarchy of laboratory rats on behavioural measures of welfare. PLoS One 12:e0185135. 10.1371/journal.pone.0185135 28926644 PMC5605050

[B5] Basner M, Babisch W, Davis A, Brink M, Clark C, Janssen S, Stansfeld S (2014) Auditory and non-auditory effects of noise on health. Lancet 383:1325–1332. 10.1016/S0140-6736(13)61613-X 24183105 PMC3988259

[B6] Bekkedal MYV, Rossi J, Panksepp J (2011) Human brain EEG indices of emotions: delineating responses to affective vocalizations by measuring frontal theta event-related synchronization. Neurosci Biobehav Rev 35:1959–1970. 10.1016/j.neubiorev.2011.05.001 21596060

[B7] Benjamini Y, Hochberg Y (1995) Controlling the false discovery rate: a practical and powerful approach to multiple testing. J R Stat Soc Series B Stat Methodol 57:289–300. 10.1111/j.2517-6161.1995.tb02031.x

[B8] Bohil CJ, Alicea B, Biocca FA (2011) Virtual reality in neuroscience research and therapy. Nat Rev Neurosci 12:752–762. 10.1038/nrn3122 22048061

[B9] Bower I, Tucker R, Enticott P (2019) Impact of built environment design on emotion measured via neurophysiological correlates and subjective indicators: a systematic review. J Environ Psychol 66:101344. 10.1016/j.jenvp.2019.101344

[B10] Bradley MM, Lang PJ (1994) Measuring emotion: the self-assessment manikin and the semantic differential. J Behav Ther Exp Psychiatry 25:49–59. 7962581 10.1016/0005-7916(94)90063-9

[B11] Brouwer GJ, van Ee R, Schwarzbach J (2005) Activation in visual cortex correlates with the awareness of stereoscopic depth. J Neurosci 25:10403–10413. 10.1523/JNEUROSCI.2408-05.2005 16280579 PMC6725836

[B12] Camm A, Malik M, Bigger J, Breithardt G, Cerutti S, Cohen R (1996) Heart rate variability: standards of measurement, physiological interpretation and clinical use. Task Force of the European Society of Cardiology and the North American Society of Pacing and Electrophysiology. Circulation 93:1043–1065. 8598068

[B13] Carl C, Açık A, König P, Engel AK, Hipp JF (2012) The saccadic spike artifact in MEG. Neuroimage 59:1657–1667. 21963912 10.1016/j.neuroimage.2011.09.020

[B14] Caspi A, Moffitt TE (2006) Gene–environment interactions in psychiatry: joining forces with neuroscience. Nat Rev Neurosci 7:583–590. 10.1038/nrn1925 16791147

[B15] Coan JA, Allen JJB (2004) Frontal EEG asymmetry as a moderator and mediator of emotion. Biol Psychol 67:7–49. 10.1016/j.biopsycho.2004.03.002 15130524

[B16] Coburn A, Vartanian O, Kenett YN, Nadal M, Hartung F, Hayn-Leichsenring G, Navarrete G, González-Mora JL, Chatterjee A (2020) Psychological and neural responses to architectural interiors. Cortex 126:217–241. 10.1016/j.cortex.2020.01.009 32092492

[B17] Damasio AR (1998) Emotion in the perspective of an integrated nervous system. Brain Res Brain Res Rev 26:83–86. 10.1016/S0165-0173(97)00064-7 9651488

[B18] Davidson RJ (1992) Anterior cerebral asymmetry and the nature of emotion. Brain Cogn 20:125–151. 10.1016/0278-2626(92)90065-T 1389117

[B19] Davidson RJ (2004) What does the prefrontal cortex “do” in affect: perspectives on frontal EEG asymmetry research. Biol Psychol 67:219–33233. 10.1016/j.biopsycho.2004.03.008 15130532

[B20] Delorme A, Makeig S (2004) EEGLAB: an open source toolbox for analysis of single-trial EEG dynamics including independent component analysis. J Neurosci Methods 134:9–21. 10.1016/j.jneumeth.2003.10.009 15102499

[B21] Demaree HA, Everhart DE, Youngstrom EA, Harrison DW (2005) Brain lateralization of emotional processing: historical roots and a future incorporating “dominance”. Behav Cogn Neurosci Rev 4:3–20. 10.1177/1534582305276837 15886400

[B22] Ekman P, Davidson R (1993) Voluntary smiling changes regional brain activity. Psychol Sci 4:342–345. 10.1111/j.1467-9280.1993.tb00576.x

[B23] Evans GW, Schroeder A, Lepore SJ (1996) The role of interior design elements in human responses to crowding. J Pers Soc Psychol 70:41–46. 10.1037/0022-3514.70.1.41

[B24] Fanger PO (1970) Thermal comfort. Analysis and applications in environmental engineering. Copenhagen: Danish Technical Press.

[B25] Faul F, Erdfelder E, Lang AG, Buchner A (2007) G*Power 3: a flexible statistical power analysis program for the social, behavioral, and biomedical sciences. Behav Res Methods 39:175–191. 10.3758/bf03193146 17695343

[B26] Fich LB, Jönsson P, Kirkegaard PH, Wallergård M, Garde AH, Hansen Å (2014) Can architectural design alter the physiological reaction to psychosocial stress? A virtual TSST experiment. Physiol Behav 135:91–97. 10.1016/j.physbeh.2014.05.034 24907691

[B27] Fitzgibbon SP, Pope KJ, Mackenzie L, Clark CR, Willoughby JO (2004) Cognitive tasks augment gamma EEG power. Clin Neurophysiol 115:1802–1809. 10.1016/j.clinph.2004.03.009 15261859

[B28] Genovese CR (2015) False discovery rate control. In: Brain mapping (Toga AW, ed), pp 501–507. Waltham: Academic Press.

[B29] Hagemann D, Naumann E, Becker G, Maier S, Bartussek D (1998) Frontal brain asymmetry and affective style: a conceptual replication. Psychophysiology 35:372–388. 9643052

[B30] Hagemann D, Waldstein SR, Thayer JF (2003) Central and autonomic nervous system integration in emotion. Brain Cogn 52:79–87. 12812807 10.1016/s0278-2626(03)00011-3

[B31] Hipp JF, Siegel M (2013) Dissociating neuronal gamma-band activity from cranial and ocular muscle activity in EEG. Front Hum Neurosci 7:338. 23847508 10.3389/fnhum.2013.00338 PMC3706727

[B32] Hoisington AJ, Stearns-Yoder KA, Schuldt SJ, Beemer CJ, Maestre JP, Kinney KA, Postolache TT, Lowry CA, Brenner LA (2019) Ten questions concerning the built environment and mental health. Build Environ 155:58–69. 10.1016/j.buildenv.2019.03.036

[B33] Hyvärinen A, Oja E (2000) Independent component analysis: algorithms and applications. Neural Netw 13:411–430. 10.1016/S0893-6080(00)00026-5 10946390

[B34] Janssen H, Bernhardt J, Walker FR, Spratt NJ, Pollack M, Hannan AJ, Nilsson M (2018) Environmental enrichment: neurophysiological responses and consequences for health. In: Oxford textbook of nature and public health: the role of nature in improving the health of a population (van den Bosch M and Bird W, eds). Oxford: Oxford University Press.

[B35] Jensen O, Kaiser J, Lachaux J-P (2007) Human gamma-frequency oscillations associated with attention and memory. Trends Neurosci 30:317–324. 17499860 10.1016/j.tins.2007.05.001

[B36] Johnson JA (2014) Measuring thirty facets of the Five Factor Model with a 120-item public domain inventory: development of the IPIP-NEO-120. J Res Person 51:78–89. 10.1016/j.jrp.2014.05.003

[B37] Kalantari S, Rounds J, Kan J, Tripathi V, Cruz-Garza J (2021) Comparing physiological responses during cognitive tests in virtual environments vs. in identical real-world environments. Sci Rep 11:10227. 10.1038/s41598-021-89297-y 33986337 PMC8119471

[B38] Kassam KS, Mendes WB (2013) The effects of measuring emotion: physiological reactions to emotional situations depend on whether someone is asking. PLoS One 8:e64959. 10.1371/journal.pone.0064959 23785407 PMC3680163

[B39] Keil A, Müller MM, Ray WJ, Gruber T, Elbert T (1999) Human gamma band activity and perception of a gestalt. J Neurosci 19:7152–7161. 10.1523/JNEUROSCI.19-16-07152.1999 10436068 PMC6782859

[B40] LeBlanc J, Ducharme MB, Pasto L, Thompson M (2003) Response to thermal stress and personality. Physiol Behav 80:69–74. 10.1016/s0031-9384(03)00225-7 14568309

[B41] Levenson RW (1988) Emotion and the autonomic nervous system: a prospectus for research on autonomic specificity. In: Social psychophysiology and emotion: theory and clinical applications, pp 17–42. Oxford: Wiley.

[B42] Lopez RB, Denny BT, Fagundes CP (2018) Neural mechanisms of emotion regulation and their role in endocrine and immune functioning: a review with implications for treatment of affective disorders. Neurosci Biobehav Rev 95:508–514. 10.1016/j.neubiorev.2018.10.019 30385251

[B43] McCrae RR, John OP (1992) An introduction to the five-factor model and its applications. J Pers 60:175–215. 10.1111/j.1467-6494.1992.tb00970.x 1635039

[B44] McDonald MW, Hayward KS, Rosbergen ICM, Jeffers MS, Corbett D (2018) Is environmental enrichment ready for clinical application in human post-stroke rehabilitation? Front Behav Neurosci 12:135–135. 10.3389/fnbeh.2018.00135 30050416 PMC6050361

[B45] Mehrabian A (1996) Pleasure-arousal-dominance: a general framework for describing and measuring individual differences in temperament. Curr Psychol 14:261–292. 10.1007/BF02686918

[B46] Müller MM, Keil A, Gruber T, Elbert T (1999) Processing of affective pictures modulates right-hemispheric gamma band EEG activity. Clin Neurophysiol 110:1913–1920. 10.1016/S1388-2457(99)00151-0 10576487

[B47] Müller MM, Gruber T, Keil A (2000) Modulation of induced gamma band activity in the human EEG by attention and visual information processing. Int J Psychophysiol 38:283–299. 11102668 10.1016/s0167-8760(00)00171-9

[B48] Mutanen TP, Metsomaa J, Liljander S, Ilmoniemi RJ (2018) Automatic and robust noise suppression in EEG and MEG: the SOUND algorithm. Neuroimage 166:135–151. 10.1016/j.neuroimage.2017.10.021 29061529

[B49] Nithianantharajah J, Hannan A (2006) Enriched environments, experience-dependent plasticity and disorders of the nervous system. Nat Rev Neurosci 7:697–709. 10.1038/nrn1970 16924259

[B50] Oostenveld R, Fries P, Maris E, Schoffelen J-M (2011) FieldTrip: open source software for advanced analysis of MEG, EEG, and invasive electrophysiological data. Comput Intell Neurosci 2011:156869. 10.1155/2011/156869 21253357 PMC3021840

[B51] Pettijohn KA, Radvansky GA (2016) Walking through doorways causes forgetting: event structure or updating disruption? Q J Exp Psychol (Hove) 69:2119–2129. 10.1080/17470218.2015.1101478 26556012

[B52] Pion-Tonachini L, Kreutz-Delgado K, Makeig S (2019) ICLabel: an automated electroencephalographic independent component classifier, dataset, and website. Neuroimage 198:181–197. 10.1016/j.neuroimage.2019.05.026 31103785 PMC6592775

[B53] Quintana DS, Heathers JAJ (2014) Considerations in the assessment of heart rate variability in biobehavioral research. Front Psychol 5:805–805. 10.3389/fpsyg.2014.00805 25101047 PMC4106423

[B54] Raskin E (1954) Architecturally speaking. New York: Reinhold.

[B55] Rousselet GA, Pernet CR, Wilcox RR (2017) Beyond differences in means: robust graphical methods to compare two groups in neuroscience. Eur J Neurosci 46:1738–1748. 10.1111/ejn.13610 28544058

[B56] Sanchez-Vives MV, Slater M (2005) Opinion: from presence to consciousness through virtual reality. Nat Rev Neurosci 6:332–339. 10.1038/nrn1651 15803164

[B57] Schneiderman N, Ironson G, Siegel SD (2005) Stress and health: psychological, behavioral, and biological determinants. Annu Rev Clin Psychol 1:607–628. 10.1146/annurev.clinpsy.1.102803.144141 17716101 PMC2568977

[B58] Seppänen OA, Fisk WJ (2004) Summary of human responses to ventilation. Indoor Air 14 [Suppl 7]:102–118. 10.1111/j.1600-0668.2004.00279.x 15330778

[B59] Seppänen OA, Fisk WJ (2006) Some quantitative relations between indoor environmental quality and work performance or health. HVAC&R Res 12:957–973. 10.1080/10789669.2006.10391446

[B60] Shaffer F, Ginsberg JP (2017) An overview of heart rate variability metrics and norms. Front Public Health 5:258–258. 10.3389/fpubh.2017.00258 29034226 PMC5624990

[B61] Silva LBd, de Souza EL, de Oliveira PAA, Andrade BJM (2019) Implications of indoor air temperature variation on the health and performance of Brazilian students. Indoor Built Environ 1420326X19878228.

[B62] Simos PG, Papanikolaou E, Sakkalis E, Sifis M (2002) Modulation of gamma-band spectral power by cognitive task complexity. Brain Topogr 14:191–196. 12002349 10.1023/a:1014550808164

[B63] Spitzer B, Haegens S (2017) Beyond the status quo: a role for beta oscillations in endogenous content (re)activation. eNeuro 4:ENEURO.0170-17.2017. 10.1523/ENEURO.0170-17.2017 PMC5539431 28785729

[B80] Standards Australia (2017) Timber and composite doors (AS 2688-2017). In: SAI Global.

[B64] Tallon-Baudry C, Bertrand O, Peronnet F, Pernier J (1998) Induced γ-band activity during the delay of a visual short-term memory task in humans. J Neurosci 18:4244–4254. 9592102 10.1523/JNEUROSCI.18-11-04244.1998 PMC6792803

[B65] Tang MF, Smout CA, Arabzadeh E, Mattingley JB (2018) Prediction error and repetition suppression have distinct effects on neural representations of visual information. Elife 7:e33123. 10.7554/eLife.33123 30547881 PMC6312401

[B66] Thayer JF, Lane RD (2000) A model of neurovisceral integration in emotion regulation and dysregulation. J Affect Disord 61:201–216. 10.1016/S0165-0327(00)00338-4 11163422

[B67] Vartanian O, Navarrete G, Chatterjee A, Fich LB, Gonzalez-Mora JL, Leder H, Modroño C, Nadal M, Rostrup N, Skov M (2015) Architectural design and the brain: effects of ceiling height and perceived enclosure on beauty judgments and approach-avoidance decisions. J Environ Psychol 41:10–18. 10.1016/j.jenvp.2014.11.006

[B68] Whittaker AL, Howarth GS, Hickman DL (2012) Effects of space allocation and housing density on measures of wellbeing in laboratory mice: a review. Lab Anim 46:3–13. 10.1258/la.2011.011049 22114068

[B69] Wolfe M (1975) Room size, group size, and density: behavior patterns in a children’s psychiatric facility. Environ Behav 7:199–224. 10.1177/001391657500700205

