# Supplementary material for: Enlarged Interior Built Environment Scale Modulates High-Frequency EEG Oscillations
Source: eNeuro. 2022 Sep 21;9(5):ENEURO.0104-22.2022. doi: 10.1523/ENEURO.0104-22.2022 (PMC9512621; doi:10.1523/ENEURO.0104-22.2022)
Supplement: Extended Data Figure 2-1 — Descriptives (mean/SD) for EEG analysis. Download Figure 2-1, DOCX file. [file enu-eN-NWR-0104-22-s03.docx]

| Condition | Resting | Small | Control | Large | Extra large |
| --- | --- | --- | --- | --- | --- |
| **EEG overall power spectral density** | | | | | |
| Delta 1 to 3 Hz (log10+3) mean / s.d. | 2.94 / .350 | 3.09 / .310 | 3.04 / .256 | 3.08 / .313 | 3.11 / .285 |
| Theta 4 to 7 Hz (log10+3) mean / s.d. | 2.36 / .320 | 2.46 / .232 | 2.44 / .223 | 2.45 / .270 | 2.47 / .274 |
| Alpha 8 to 12 Hz (log10+3) mean / s.d. | 2.13 / .435 | 2.09 / .346 | 2.07 / .355 | 2.09 / .352 | 2.09 / .364 |
| Beta 13 to 29 Hz (log10+3) mean / s.d. | 1.67 / .283 | 1.70 / .276 | 1.71 / .287 | 1.72 / .296 | 1.74 / .276 |
| Low Gamma 30 to 45 (log10+3) mean / s.d. | 1.18 / .227 | 1.32 / .255 | 1.28 / .258 | 1.33 / .287 | 1.36 / .271 |
| High Gamma 55 to 70 (log10+3) mean / s.d. | .695 / .282 | .916 / .321 | .838 / .323 | .870 / .321 | .892 / .319 |
| **EEG frontal hemispheric lateralization (Left – Right / Right + Left) * 100)** | | | | | |
| Theta 4 to 7 Hz (log10+301) mean / s.d | 2.40 / .070 | .243 / .058 | 2.43 / .052 | 2.43 / .064 | 2.43 / .080 |
| Alpha 8 to 12 Hz (log10+350) mean / s.d | 2.45 / .054 | 2.44 / .054 | 2.44 / .051 | 2.45 / .049 | 2.44 / .055 |
| **EEG hemispheric lateralization (Left – Right / Right + Left) * 100)** | | | | | |
| Low Gamma 30 to 45 (log10+575) mean / s.d | 2.69 / .031 | 2.69 / .023 | 2.69 / .031 | 2.68 / .024 | 2.69 / .030 |
| High Gamma 55 to 70 (log10+575) mean / s.d | 2.69 / .026 | 2.69 / .026 | 2.69 / .030 | 2.68 / .030 | 2.69 / .031 |
| **EEG frontal midline power spectral density** | | | | | |
| Theta 4 to 7 Hz (log10+3) mean / s.d | 2.66 / .394 | 2.75 / .364 | 2.73 / .353 | 2.74 / .354 | 2.77 / .374 |
| Alpha 8 to 12 Hz (log10+3) mean / s.d | 2.29 / .489 | 2.19 / .385 | 2.19 / .385 | 2.23 / .409 | 2.19 / .393 |
| Low Gamma 30 to 45 (log10+3) mean / s.d | 1.08 / .237 | 1.20 / .258 | 1.17 / .256 | 1.14 / .243 | 1.22 / .246 |
| High Gamma 55 to 70 (log10+3) mean / s.d | .565 / .340 | .711 / .330 | .688 / .343 | .673 / .315 | .755 / .336 |

**Figure 2-1.** Descriptives (mean / standard deviation) for EEG analysis.
