# Supplementary material for: Enlarged Interior Built Environment Scale Modulates High-Frequency EEG Oscillations
Source: eNeuro. 2022 Sep 21;9(5):ENEURO.0104-22.2022. doi: 10.1523/ENEURO.0104-22.2022 (PMC9512621; doi:10.1523/ENEURO.0104-22.2022)
Supplement: Extended Data Figure 2-2 — Statistical significance values for EEG analysis. Note: power spectral density, frontal midline, and θ and α lateralization statistics are derived from parametric one-way repeated measures ANOVAs. p, p value; FDR, FDR correction. Download Figure 2-2, DOCX file. [file enu-eN-NWR-0104-22-s04.docx]

| Condition | Resting | | | | Small | | | Control  Large | | | Large |
| --- | --- | --- | --- | --- | --- | --- | --- | --- | --- | --- | --- |
| Comparison | Small | Control | Large | Extra large | Control | Large | Extra large | Large | Extra large | Extra large | |
| **EEG power spectral density** | | | | | | | | | | | |
| Delta 1 to 3 Hz (log10+3) P / P ^FDR^ | **<.001 / <.001** | **<.001/ <.001** | **<.001 / <.001** | **<.001/ <.001** | .368 / .420 | .271 / .387 | .378 / .420 | .912 / .912 | .082 / .137 | .059 / .118 | |
| Theta 4 to 7 Hz (log10+3) P / P ^FDR^ | **<.001 / <.001** | **<.001 / <.001** | **<.001 / <.001** | **<.001 / <.001** | .458 / 509 | .930 / .930 | .247 / .411 | .391 / .489 | .106 / .212 | .333 / .476 | |
| Alpha 8 to 12 Hz (log10+3) P / P ^FDR^ | .017 / .070 | **.003 / .030** | .024 / .070 | .028 / .070 | .395 / .564 | .506 / .633 | .587 / .652 | .096 / .192 | .195 / .325 | .910 / .091 | |
| Beta 13 to 29 Hz (log10+3) P / P ^FDR^ | **.007 / .016** | **.003 / .015** | **.008 / .016** | **<.001 / <.001** | .937 / .937 | .695 / .772 | **.031 / .044** | .658 / .772 | **.011 / .018** | **.007 / .016** | |
| Low Gamma 30 to 45 (log10+3) P / P ^FDR^ | **<.001 / <.001** | **<.001 / <.001** | **<.001 / <.001** | **<.001 / <.001** | .353 / .441 | .401 / .446 | .319 / .441 | .969 / .969 | .058 / .116 | .130 / .217 | |
| High Gamma 55 to 70 (log10+3) P / P ^FDR^ | **<.001 / <.001** | **<.001 / <.001** | **<.001 / <.001** | **<.001 / <.001** | .065 / .108 | .763 / .763 | .466 / .518 | .207 / .296 | .040 / .080 | .297 / .371 | |
| **EEG frontal hemispheric lateralization (Left – Right / Right + Left) * 100)** | | | | | | | | | | | |
| Theta 4 to 7 Hz (log10+301) *P* / *P* ^FDR^ | **<.001 / <.001** | **<.001 / <.001** | **<.001 / <.001** | **<.001 / <.001** | .856 / .930 | .659 / .928 | .742 / .928 | .536 / .928 | .665 / .928 | .930 / .930 | |
| Alpha 8 to 12 Hz (log10+350) *P* / *P* ^FDR^ | .016 / .080 | .015 / .080 | .084 / .174 | .120 / .174 | .550 / .611 | .108 / .174 | .034 / .113 | .174 / .218 | .122 / .174 | .726 / .726 | |
| **EEG hemispheric lateralization (Left – Right / Right + Left) * 100)** | | | | | | | | | | | |
| Low Gamma 30 to 45 (log10+575) *P* / *P* ^FDR^ | .724 / 872 | .642 / .872 | .168 / .420 | .533 / .872 | .785 / .872 | .037 / .123 | .690 / .872 | .020 / .100 | .909 / .909 | **.005 / .050** | |
| High Gamma 55 to 70 (log10+575) *P* / *P* ^FDR^ | .936 / .936 | .282 / .403 | .186 / .403 | .841 / .934 | .281 / .403 | .198 / .403 | .778 / .934 | .026 / .260 | .224 / .403 | .190 / .403 | |
| **EEG frontal midline power spectral density** | | | | | | | | | | | |
| Theta 4 to 7 Hz (log10+3) *P* / *P* ^FDR^ | **<.001 / <.001** | **<.001 / <.001** | **<.001 / <.001** | **<.001 / <.001** | .708 / .790 | .962 / .962 | .618 / .790 | .711 / .790 | .456 / .790 | .594 / .790 | |
| Alpha 8 to 12 Hz (log10+3) *P* / *P* ^FDR^ | .026 / .130 | .009 / .090 | .053 / .150 | .060 / .150 | .685 / .761 | .345 / .493 | .419 / .524 | .120 / .240 | .280 / .467 | .812 / .812 | |
| Low Gamma 30 to 45 (log10+3) *P* / *P* ^FDR^ | **<.001 / <.001** | **<.001 / <.001** | **<.001 / <.001** | **<.001 / <.001** | .610 / .610 | .150 / .188 | .073 / .104 | .313 / .348 | **.012 / .020** | **.002 / .004** | |
| High Gamma 55 to 70 (log10+3) *P* / *P* ^FDR^ | **<.001 / <.001** | **<.001 / <.001** | **<.001 / <.001** | **<.001 / <.001** | .212 / .265 | .197 / .265 | .267 / .297 | .850 / .850 | **.027 / .045** | **.024 / .045** | |

**Figure 2-2.** Statistical significance values for EEG analysis. Note: Power spectral density, frontal midline and theta and alpha lateralisation statistics are derived from parametric one-way repeated measures ANOVA’s. *P* = P value, ^FDR^ = False discovery rate correction.
