# Supplementary material for: Enlarged Interior Built Environment Scale Modulates High-Frequency EEG Oscillations
Source: eNeuro. 2022 Sep 21;9(5):ENEURO.0104-22.2022. doi: 10.1523/ENEURO.0104-22.2022 (PMC9512621; doi:10.1523/ENEURO.0104-22.2022)
Supplement: Extended Data Figure 4-1 — Descriptives (mean/SD) for physiological analysis. Download Figure 4-1, DOCX file. [file enu-eN-NWR-0104-22-s06.docx]

| Condition | Resting | Small | Control | Large | Extra large |
| --- | --- | --- | --- | --- | --- |
| HRV RMSSD (log10) mean / s.d. | 1.54 / .210 | 1.50 / .223 | 1.49 / .204 | 1.52 / .232 | 1.50 / .234 |
| HRV SDRR (log10) mean / s.d. | 1.68 / .165 | 1.63 / .189 | 1.64 / .179 | 1.63 / .180 | 1.64 / .188 |
| Resp mean (no transform) mean / s.d. | 17.9 / 3.94 | 18.5 / 3.48 | 18.5 / 2.92 | 18.6 / 3.71 | 18.6 / 3.64 |
| Resp mx-mn (no transform) mean / s.d. | 1.41 / .329 | 1.44 / .287 | 1.48 / .243 | 1.50 / .208 | 1.45 / .314 |
| SCR mean (log10) mean / s.d. | 1.41 / .032 | 1.42 / .031 | 1.41 / .034 | 1.41 / .032 | 1.41 / .021 |
| SCR mx-mn (log10) mean / s.d. | .337 / .543 | .627 / .391 | .662 / .374 | .681 / .371 | .695 / .288 |

**Figure 4-1.** Descriptives (mean / standard deviation) for physiological analysis.
