# Supplementary material for: Enlarged Interior Built Environment Scale Modulates High-Frequency EEG Oscillations
Source: eNeuro. 2022 Sep 21;9(5):ENEURO.0104-22.2022. doi: 10.1523/ENEURO.0104-22.2022 (PMC9512621; doi:10.1523/ENEURO.0104-22.2022)
Supplement: Extended Data Figure 4-2 — Statistical significance values for physiological measures. Note: statistics are derived from one-way repeated measures ANOVAs. p, p value; FDR, FDR correction. Download Figure 4-2, DOCX file. [file enu-eN-NWR-0104-22-s07.docx]

| Condition | Resting | | | | Small | | | Control | | Large |
| --- | --- | --- | --- | --- | --- | --- | --- | --- | --- | --- |
| Comparison | Small | Control | Large | Extra large | Control | Large | Extra large | Large | Extra large | Extra large |
| HRV RMSSD (log10) *P* / *P* ^FDR^ | .019 / .063 | **.001 / .001** | .063 / .157 | **.007 / .035** | .348 / .536 | .571 / .634 | .750 / .750 | .178 / .356 | .375 / .536 | .435 / .544 |
| HRV SDRR (log10) *P* / *P* ^FDR^ | .010 / .065 | .130 / .325 | .013 / .065 | .027 / .090 | .216 / .432 | .825 / .825 | .513 / .716 | .309 / .515 | .573 / .716 | .667 / .741 |
| Resp mean (no transform) *P* / *P* ^FDR^ | .136 / .375 | .150 / .375 | .019 / .190 | .052 / .260 | .722 / .802 | .557 / .784 | .627 / .784 | .343 / .597 | .358 / .597 | .892 / .892 |
| Resp mx-mn (no transform) *P* / *P* ^FDR^ | .263 / .658 | .205 / .658 | .117 / .585 | .104 / .911 | .797 / .911 | .622 / .911 | .614 / .911 | .719 / .911 | .820 / .911 | .998 / .998 |
| SCR mean (log10) *P* / *P* ^FDR^ | .071 / .410 | .123 / .410 | .122 / .410 | .317 / .528 | .454 / .567 | .527 / .586 | .173 / .433 | .927 / .927 | .390 / .557 | .291 / .528 |
| SCR mx-mn (log10) *P* / *P* ^FDR^ | **< .001 / < .001** | **< .001 / < .001** | **< .001 / < .001** | **< .001 / < .001** | .980 / .980 | .417 / .763 | .681 / .769 | .458 / .763 | .692 / .769 | .622 / .769 |

**Figure 4-2.** Statistical significance values for physiological measures. Note: Statistics are derived from one-way repeated measures ANOVA’s. *P* = P value, ^FDR =^ False discovery rate correction.
